# Spatially Resolved Multi-Omics Reveals Brain–Kidney Compartmentalization and Region-Specific Molecular Reprogramming After Acute Nicotine Exposure

**DOI:** 10.3390/metabo16090657

**Published:** 2026-09-08

**Authors:** Qian Li, Lutao Xu, Mingyu Zhu, Gaoge Wang, Yu Bai, Huan Chen, Hongwei Hou

**Affiliations:** 1 Beijing Life Science Academy, Beijing 102299, China; noone639@163.com (Q.L.);; 2 Key Laboratory of Tobacco Biological Effects, China National Tobacco Quality Supervision & Test Center, Zhengzhou 450001, China; 3 College of Chemistry and Molecular Engineering, Peking University, Beijing 100871, China

**Keywords:** mass spectrometry imaging, spatial multi-omics, spatial proteomics, metabolomics, nicotine

## Abstract

Background: Traditional bulk tissue analyses obscure the precise spatial compartmentalization of nicotine and its molecular effects within individual anatomical regions. This study aimed to develop and apply a high-resolution spatial multi-omics framework to characterize the localized disposition and functional responses induced by an acute nicotine challenge. Methods: We established a spatial multi-omics framework integrating matrix-assisted laser desorption/ionization time-of-flight mass spectrometry imaging (MALDI-TOF MSI), air-flow-assisted desorption electrospray ionization mass spectrometry imaging (AFADESI-MSI), laser microdissection (LMD)-based microscale data-independent acquisition (microDIA) proteomics, and targeted LC-MS/MS. This platform was used to analyze the kidney and five brain regions in rats subjected to an acute nicotine challenge following an adaptation regimen. Results: Spatial mapping revealed distinct peripheral and central distribution patterns: nicotine, cotinine, and nornicotine accumulated predominantly in the renal cortex and medulla, whereas their distribution in the brain is region-dependent, with a prominent 3-hydroxycotinine signal in the olfactory bulb. Avoiding tissue homogenization enabled these spatial distributions to be linked to localized functional responses. The striatal dopamine/DOPAC axis showed the strongest acute neurochemical response, consistent with increased dopamine turnover. Spatial metabolomics further demonstrated robust, region-specific metabolic reprogramming, with the hippocampus showing the greatest metabolic variance. LMD-resolved proteomics identified protein-level changes, particularly in the olfactory bulb and thalamus. Cross-omics revealed coordinated alterations in purine, pyrimidine, glycerophospholipid, and alanine/aspartate/glutamate metabolism, with the thalamus showing the greatest extensive metabolite–protein concordance. Conclusions: These findings characterize acute nicotine exposure as a spatially compartmentalized process involving renal handling, region-specific brain distribution, and localized molecular response programs.

## 1. Introduction

Nicotine is the principal psychoactive constituent of tobacco products and electronic nicotine-delivery systems. Because of its small molecular size and high lipophilicity, nicotine rapidly crosses the blood–brain barrier and modulates dopamine, acetylcholine, glutamate, and GABA signaling networks, thereby affecting reward, attention, stress responses, and addictive behaviors [1,2,3]. However, acute nicotine exposure is not confined to the central nervous system; it is a highly coordinated systemic process involving central neuropharmacological effects and peripheral metabolism and clearance [4,5,6,7].

The brain and kidney are biologically distinct components of the systemic response to nicotine, and both exhibit pronounced anatomical and functional heterogeneity. Brain regions and nuclei differ substantially in receptor distribution, neurotransmitter activity, and basal energy demand, whereas the kidney mediates xenobiotic transport, concentration, and elimination across distinct cortical and medullary compartments. Accordingly, acute nicotine exposure is unlikely to produce a spatially uniform response, but rather region- and compartment-dependent molecular changes. Conventional studies of xenobiotic exposure have largely relied on bulk-tissue homogenates or biofluid measurements [8]. Although these approaches are useful for assessing overall exposure, tissue homogenization averages signals across functionally distinct microregions and may therefore obscure localized molecular responses [9]. To address this spatial limitation, mass spectrometry imaging (MSI) and spatially resolved multi-omics provide complementary analytical approaches. MALDI-TOF MSI enables the direct in situ visualization of xenobiotics and their metabolites, whereas AFADESI-MSI provides broad profiling of endogenous metabolic remodeling without disrupting the tissue matrix [10,11,12,13]. In addition, coupling laser microdissection (LMD) with downstream LC-MS/MS or microDIA proteomics enables molecular features to be assigned to defined anatomical microregions [14,15].

In this study, we developed an integrated spatial multi-omics framework to characterize responses to an acute nicotine challenge across two renal compartments and five functionally distinct brain regions the olfactory bulb (OB); prefrontal cortex (PFC); striatum (STR); hippocampus (HIPP); and thalamus (THA). By strategically combining MALDI-TOF and AFADESI-MSI with LMD-resolved microDIA proteomics and targeted LC-MS/MS, we reduced the spatial averaging inherent in conventional tissue homogenization. We examined whether acute nicotine exposure produces compartment-specific distributions across the brain and kidney, how these spatial patterns are associated with local neurotransmitter changes, and whether the metabolomic and proteomic responses converge on shared functional modules. This spatially resolved approach was intended to provide characterize acute xenobiotic exposure as an anatomically organized molecular response rather than a uniform systemic perturbation.

## 2. Materials and Methods

### 2.1. Reagents and Materials

Nicotine, its major nicotine metabolites, and neurotransmitter standards were obtained primarily from MedChemExpress (MCE, Monmouth Junction, NJ, USA) and used to develop targeted assays, confirm retention times, construct calibration curves, and correct quantitative measurements. LC-MS-grade methanol, acetonitrile, and formic acid were obtained from Thermo Fisher Scientific (Shanghai, China). Ultrapure water was prepared using a Milli-Q Academic system (Millipore, Bedford, MA, USA). CMC-Na embedding medium, MALDI matrices, calibration reagents (Thermo Fisher Scientific, Waltham, MA, USA), conductive or positively charged slides (CITOTEST Labware Manufacturing Co., Ltd., Haimen, Jiangsu, China), and routine analytical reagents were prepared as required for MSI, laser microdissection (Leica Biosystems, Danvers, MA, USA), and LC-MS/MS. Unless otherwise specified, reagents were of analytical grade or MS grade and were stored according to the suppliers’ recommendations.

### 2.2. Animal Treatment and Tissue Collection

**Figure 1:**
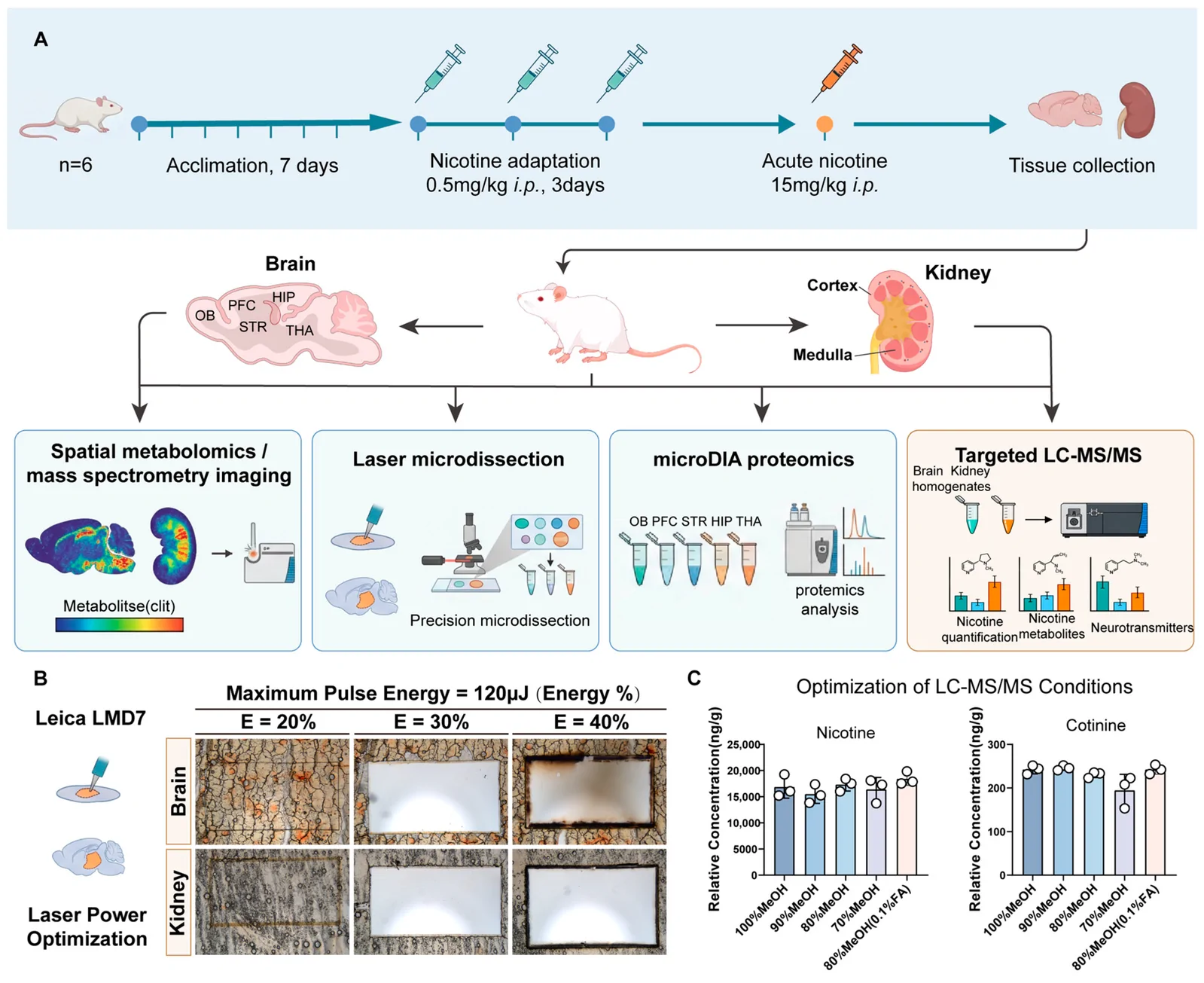
Study design and spatial multi-omics workflow for acute nicotine exposure. (**A**) Experimental timeline and tissue-processing scheme. After acclimation, rats in the nicotine-treatment arm received low-dose nicotine adaptation (0.5 mg/kg, i.p., once daily for 3 days) followed by an acute nicotine challenge (15 mg/kg, i.p.) before rapid collection of brain and kidney tissues; vehicle-treated animals underwent the corresponding control schedule. Brain regions comprised the olfactory bulb (OB), prefrontal cortex (PFC), striatum (STR), hippocampus (HIPP), and thalamus (THA), whereas kidney compartments comprised the cortex and medulla. The collected tissues were analyzed by complementary spatial and microregion-based platforms, including MALDI-TOF/AFADESI mass spectrometry imaging (MSI) for in situ metabolite mapping, laser microdissection (LMD) for anatomical sampling, targeted LC-MS/MS for nicotine, nicotine metabolites, and neurotransmitters, and microDIA proteomics for low-input microregion protein profiling. (**B**) Optimization of Leica LMD7 cutting energy in brain and kidney sections. Compared with incomplete cutting at low energy and greater edge carbonization at high energy, 30% laser energy produced continuous cutting boundaries with limited thermal damage and was used for subsequent microdissection. (**C**), Extraction-solvent optimization for targeted LC-MS/MS analysis of LMD-collected microregions. The 80% methanol/0.1% formic acid condition provided consistent recovery of nicotine and cotinine and was selected for downstream targeted assays.

Male Sprague–Dawley rats (approximately 280 ± 25 g) were purchased from Charles River Laboratories (Beijing, China) and randomly assigned to 2 groups (*n* = 6 per group, 12 animals in total). Only male rats were selected exclusively to minimize biological variance introduced by cycling female hormones (estrogen/progesterone), which heavily fluctuate baseline dopamine turnover and striatal receptor sensitivity. Randomization was used to allocate experimental units to these groups. The randomization sequence was generated using the standard random number generator in Microsoft Excel. Rats were housed under specific pathogen-free conditions at 24 ± 2 °C and 60% ± 5% humidity under a 12 h light/dark cycle, with food and water available ad libitum. Potential confounders were addressed through a structured experimental design. Group allocation and the sequence of treatments/measurements were fully randomized to prevent order-related bias. To mitigate environmental variance, all cages were maintained under identical, strictly controlled laboratory conditions (constant temperature, humidity, and a 12 h light/dark cycle) on the same shelf levels of the housing racks. Additionally, the order of treatments, sample collections, and subsequent measurements was fully randomized across all groups to eliminate time-of-day effects or batch bias. All assessments were conducted by investigators blinded to the group allocations. All animal procedures were approved by the Laboratory Animal Management and Ethics Committee of the China National Tobacco Quality Supervision and Test Center (Approval No.: CTQTC-SYXK-2025003). During the treatment process, we used high-quality, fine-sized needles for subcutaneous injections to reduce acute tissue damage. After acclimation, rats were randomly assigned to experimental and control groups. The nicotine group received 0.5 mg/kg nicotine intraperitoneally for three consecutive days as a low-dose adaptation phase, followed by a 15 mg/kg acute nicotine challenge; the 15 mg/kg challenge as an acute toxicological stress model was aimed at eliciting maximal microregional capacity for subsequent analysis, such as metabolomics and proteomics analysis; controls received vehicle. Brain and kidney tissues were rapidly collected under deep terminal anesthesia using 2.5% tribromoethanol (250 mg/kg, i.p.; T48402, Sigma-Aldrich, St. Louis, MO, USA). After loss of pedal reflex, tissues were dissected on an ice-cold surface, flash-frozen in liquid-nitrogen-chilled isopentane, and stored at −80 °C until sectioning, microdissection, and MS analysis. Brain tissue was divided into olfactory bulb (OB), prefrontal cortex (PFC), striatum (STR), hippocampus (HIPP), and thalamus (THA), whereas kidney tissue was divided into cortex and medulla. Targeted LC-MS/MS quantification and microDIA proteomics were both performed on laser-microdissected anatomical microregions rather than on bulk homogenates. This design allows nicotine, metabolites, neurotransmitters, and protein pathway changes to be traced back to defined spatial compartments (The above process is illustrated in Figure 1). All procedures were approved by the Laboratory Animal Management and Ethics Committee of the China National Tobacco Quality Supervision and Test Center and complied with institutional animal-care requirements.

### 2.3. Cryosectioning and Tissue Preparation

Frozen brain and kidney tissues were transferred from −80 °C to −20 °C and equilibrated for approximately 6 h before sectioning. Tissue sections (10 µm) were prepared using a Leica cryostat. (Leica Biosystems, Danvers, MA, USA) Sections (*n* = 6) designated for MALDI-TOF MSI and AFADESI-MSI were mounted on conductive or positively charged slides, vacuum-dried for 15 min, and analyzed immediately or stored briefly under dry low-temperature conditions. Adjacent sections used for laser microdissection were processed with minimal washing and chemical exposure to preserve morphology while limiting molecular redistribution. HE-stained adjacent sections, anatomical atlases, and kidney morphology were used to guide ROI annotation, image registration, and microdissection boundaries.

### 2.4. MALDI-TOF Mass Spectrometry Imaging

MALDI-TOF MSI was used to map nicotine and metabolite distributions in brain and kidney sections; neurotransmitter ion images were acquired using the same in situ imaging. Dried tissue sections were uniformly coated with a MALDI matrix optimized for small-molecule detection using an automated sprayer. Matrix layer thickness and crystal homogeneity were optimized on test sections before data acquisition. Data were acquired in positive ion mode over the target *m*/*z* windows after external or internal calibration. Raster scanning was executed at a spatial resolution (pixel size) of 20 µm across tissue sections. Two-dimensional ion-intensity matrices were normalized and co-registered with adjacent HE images. MALDI-TOF MSI was used for in situ distribution assessment, whereas quantitative interpretation was based on laser-microdissected LC-MS/MS measurements. The workflow was based on established MALDI-MSI strategies for small-molecule mapping in tissues and spatial metabolite imaging in brain tissues [16,17].

### 2.5. AFADESI-MSI Spatial Metabolomics

Global spatial metabolomics and imaging of representative candidate metabolites were performed using an AFADESI-MSI platform (Beijing Viktor Technology Co., Ltd., Beijing, China) coupled to a Q Exactive mass spectrometer (Thermo Fisher Scientific, Waltham, MA, USA). The spray solvent was acetonitrile/water (8:2, *v*/*v*) delivered at 6 µL/min. The spray voltage was ±3.0 kV and the auxiliary gas pressure was 0.7 MPa. Data were acquired in positive and negative ion modes over *m*/*z* 100–1000 at 120,000 resolution. Raster scanning used a speed of 0.1 mm/s and line spacing of 0.2 mm, yielding a pixel size (spatial resolution) of 100 µm across scanned tissue areas, sufficient for ROI-level metabolic analysis of major brain regions. The needle–tissue distance was approximately 3 mm, the spray angle was approximately 60°, and the ion-transfer tube temperature was 350 °C [18,19].

### 2.6. ROI Annotation and Laser Microdissection

Brain ROIs included OB, PFC, STR, HIPP, and THA; kidney ROIs included cortex and medulla. ROIs were annotated using the Paxinos and Watson rat brain atlas, adjacent HE staining, tissue morphology, and MSI ion-image contours. Samples for targeted LC-MS/MS and microDIA proteomics were collected using a Leica LMD7 laser microdissection (Leica Biosystems, Danvers, MA, USA) system. Pilot tests compared cutting integrity, tissue release efficiency, and thermal damage under different laser energies; 30% laser energy was selected for subsequent brain-region and kidney-compartment collection. Sampling areas were kept as consistent as possible within each batch, and dissected microregions were collected directly into low-binding tubes and stored on dry ice or at −80 °C until extraction.

### 2.7. Targeted LC-MS/MS of Laser-Microdissected Microregions

**Figure 2:**
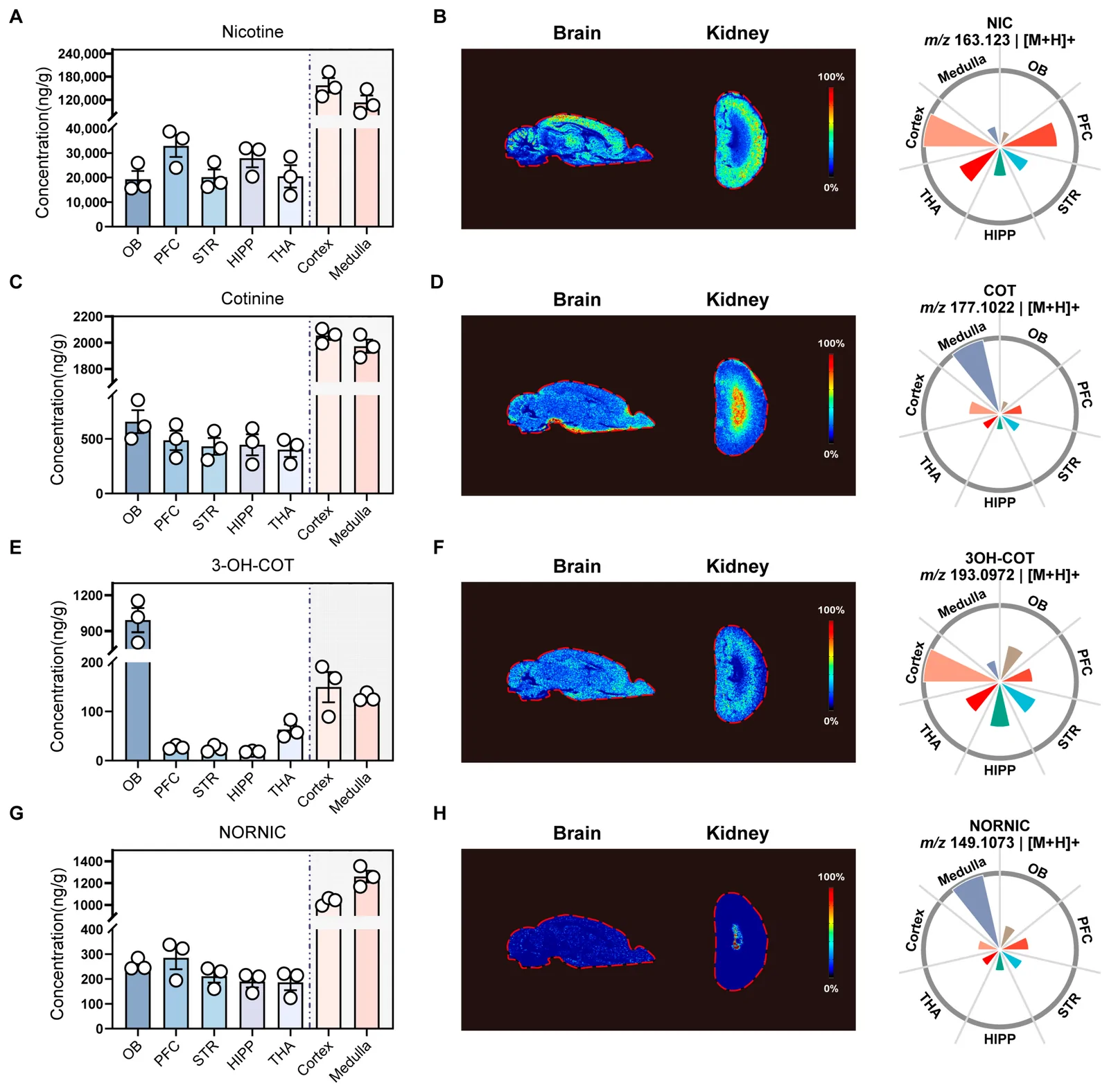
Spatial compartmentalization of nicotine and major metabolites in brain regions and kidney compartments. (**A**,**C**,**E**,**G**) Targeted LC-MS/MS quantification of nicotine, cotinine (COT), 3-hydroxycotinine (3-OH-COT), and nornicotine (NORNIC) in LMD-collected OB, PFC, STR, HIPP, and THA brain regions and in the renal cortex and medulla. Open circles denote individual microregion measurements, and bars with error bars summarize group-level concentrations. (**B**,**D**,**F**,**H**) Matched MALDI-TOF MSI ion images and polar summaries for the corresponding ions: nicotine, *m*/*z* 163.123 [M + H]^+^; COT, *m*/*z* 177.1022 [M + H]^+^; 3-OH-COT, *m*/*z* 193.0972 [M + H]^+^; and NORNIC, *m*/*z* 149.1073 [M + H]^+^. Ion images are displayed as normalized relative intensities (0–100%) while retaining tissue-level anatomical context. Nicotine, COT, and NORNIC showed prominent enrichment in renal cortex and medulla compared with brain regions, whereas 3-OH-COT exhibited a relatively strong central signal in the olfactory bulb, indicating metabolite-specific divergence superimposed on renal accumulation. Supplement: Different colors represent different brain regions.

**Figure 3:**
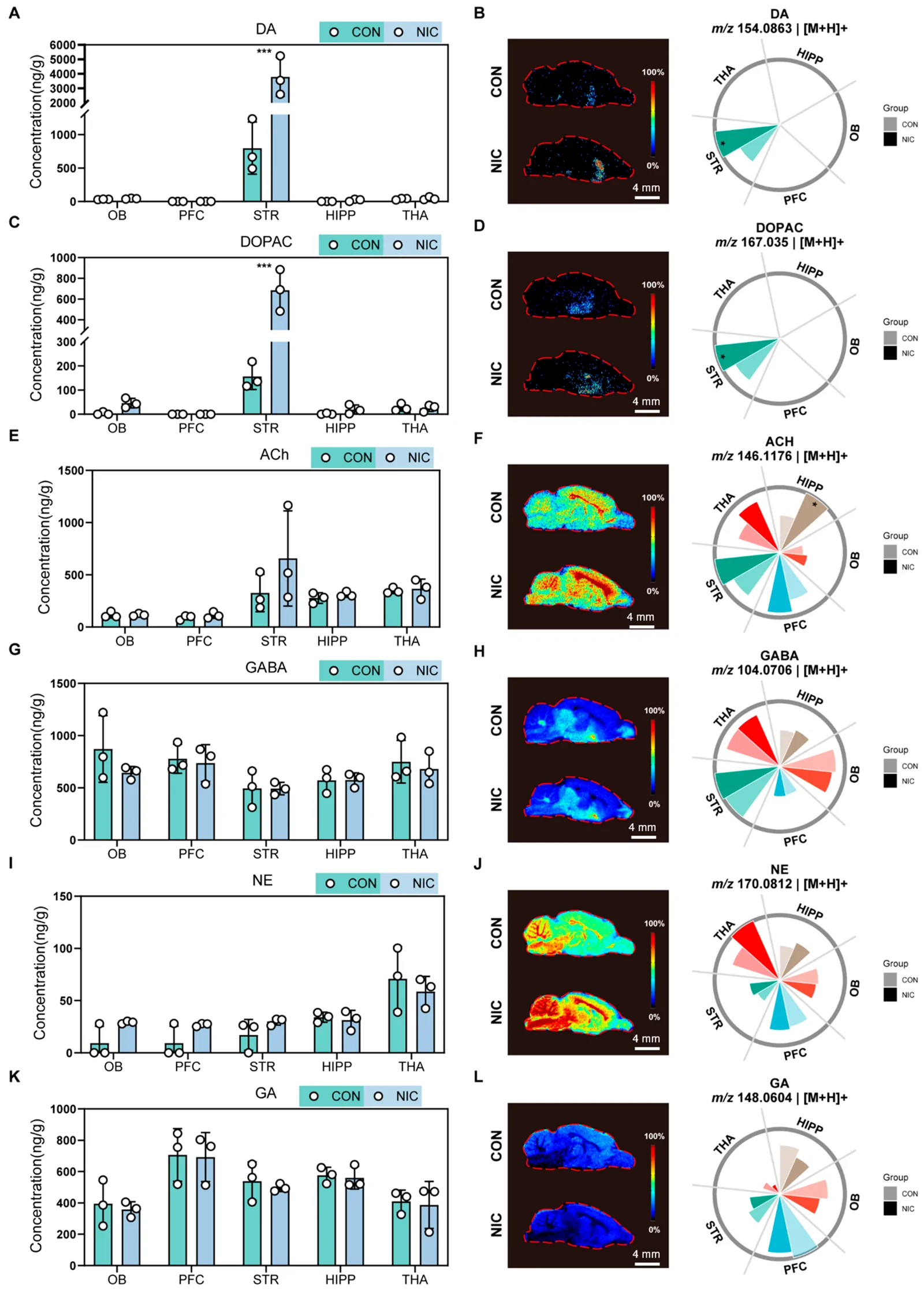
Brain-region neurochemical responses to acute nicotine exposure. (**A**,**C**,**E**,**G**,**I**,**K**) Targeted LC-MS/MS quantification of dopamine (DA), 3,4-dihydroxyphenylacetic acid (DOPAC), acetylcholine (ACh), gamma-aminobutyric acid (GABA), norepinephrine (NE), and glutamic acid (GA) in LMD-collected OB, PFC, STR, HIPP, and THA microregions from control (CON) and nicotine-treated (NIC) animals. Bars show mean values with error bars, and overlaid circles represent individual measurements. (**B**,**D**,**F**,**H**,**J**,**L**) Corresponding MSI ion images and polar summaries visualizing showing the regional distribution of each neurotransmitter-related ion in CON and NIC brain sections; the matched *m*/*z* values are provided in the panels. DA and DOPAC exhibited a pronounced striatal bias and nicotine-associated increases, consistent with rapid activation of dopamine turnover in STR, whereas ACh, GABA, NE, and GA showed smaller or more region-dependent changes. Statistical significance was assessed by comparing NIC with CON within the indicated region; * *p* < 0.05, *** *p* < 0.001. Supplement: Different colors represent different brain regions. Within the same color scheme, darker shades represent the Nic group, while lighter shades represent the Con group.

After microdissection of brain tissue and kidney regions (*n* = 3), the samples were mixed with an 80% methanol–water solution containing 0.1% formic acid and an appropriate amount of zirconia beads, homogenized at 55 Hz for 5 min at 4 °C, and then centrifuged at 12,000 rpm for 10 min at 4 °C. The supernatant was filtered. An aliquot of 40 µL of the sample solution was accurately transferred and mixed with 10 µL of a deuterated internal standard solution containing 250 ng/mL nicotine and its metabolites or 50 ng/mL deuterated neurotransmitters. After thorough mixing, the solution was ready for HPLC–MS/MS analysis. The chromatographic and mass spectrometric conditions for nicotine and its metabolites, as well as for small-molecule neurotransmitters, are provided in Supporting Information S1, Sections S2.1, S2.2, S3.1 and S3.2, together with the method validation data. Details of the HPLC–MS/MS liquid chromatography and mass spectrometry conditions, multiple reaction monitoring (MRM) transitions, calibration curves, intra- and inter-day precision, stability, matrix effect and extraction recovery are also presented in Supporting Information S1, Sections S2.3–S2.5 and S3.3–S3.5. All quantitative bar graphs in Figure 2 and Figure 3 were generated from laser-microdissected regional samples, not from whole-tissue homogenates.

### 2.8. microDIA Proteomics of Laser-Microdissected Brain Microregions

Laser-microdissected OB, PFC, STR, HIPP, and THA samples (*n* = 3) were lysed, reduced, alkylated, and digested with trypsin for LC-MS/MS data-independent acquisition (*n* = 3). Samples were separated using a Vanquish Neo UHPLC nanoflow liquid chromatography system (Thermo Fisher Scientific, Waltham, MA, USA). Mobile phase A consisted of 0.1% formic acid in water, and mobile phase B consisted of 0.1% formic acid in acetonitrile (acetonitrile 100%). The injection mode employed a trap-and-elute dual-column configuration, where the trapping column was a PepMap Neo Trap Cartridge (300 µm × 5 mm, 5 µm; Thermo Fisher Scientific, Waltham, MA, USA) and the analytical column was an Easy-Spray™ PepMap™ Neo UHPLC column (150 µm × 15 cm, 2 µm; Thermo Fisher Scientific, Waltham, MA, USA). The temperature of the analytical column was maintained at 55 °C by an integrated column oven. The loading amount was 200 ng, the flow rate was 2.5 µL/min, the effective gradient duration was 22 min, and the total run time was 24 min. After separation by nano-flow UHPLC, the samples were analyzed using an Orbitrap Astral high-resolution mass spectrometer (Thermo Fisher Scientific, Waltham, MA, USA) operated in data-independent acquisition (DIA) mode. The detection was performed in positive ion mode. The precursor ion scan range was set to *m*/*z* 380–980. The full MS resolution was 240,000 at *m*/*z* 200, with a normalized AGC target of 500% and a maximum injection time of 5 ms. For MS/MS, DIA data acquisition was employed with 299 scanning windows, an isolation window width of 2 Th, HCD collision energy of 25%, a normalized AGC target of 500%, and a maximum injection time of 3 ms. Raw data were searched using either a library-based or library-free (directDIA) workflow, followed by peptide and protein quantification, missing value imputation, batch correction, and quality control.

Raw DIA data were processed using DIA-NN version 1.8.1 in a library-free, two-pass workflow, without the use of an external DDA-derived experimental spectral library. Protein sequence information was obtained from the UniProtKB Rattus norvegicus reference proteome (Proteome ID UP000002494, downloaded 1 May 2024). The FASTA database was subjected to in silico digestion using Trypsin/P, allowing up to one missed cleavage. Peptide lengths of 7–30 amino acids and precursor charge states of 1–4 were considered. Carbamidomethylation of cysteine was specified as a fixed modification, and N-terminal methionine excision was enabled. The precursor *m*/*z* range was restricted to 380–980 to match the experimental DIA range. For the first-pass analysis, DIA-NN generated an in silico predicted spectral library directly from the protein sequence database using its deep-learning-based prediction of fragment-ion intensities and retention times. MS1 and MS2 mass tolerances were automatically optimized and resulted in 10 ppm and 10 ppm, respectively. Following the initial DIA search, confidently detected precursors across the experimental runs were used to construct a project-specific empirical spectral library, incorporating experimentally observed precursor information and retention-time characteristics. The complete DIA dataset was subsequently reanalyzed against this refined project-specific library in the second-pass analysis, with match-between-runs (MBR) enabled to improve consistency of peptide identification and quantification across runs. Identification confidence was assessed using the target–decoy strategy implemented in DIA-NN. Decoy precursor sequences were generated internally by DIA-NN and used together with target identifications to estimate identification-level q-values. Peptide precursor identifications were filtered at a false discovery rate (FDR) of ≤1% (Q.Value ≤ 0.01), and protein-group identifications were additionally controlled at ≤1% FDR (PG.Q.Value ≤ 0.01). Only protein groups satisfying these identification-confidence criteria were retained for downstream quantitative analysis.

Protein abundance was quantified using the MaxLFQ algorithm implemented in DIA-NN. Cross-run quantitative variation was minimized using DIA-NN retention-time-dependent normalization and the Robust LC quantification strategy. Because normalized quantitative values generated by DIA-NN were used for downstream analysis, no additional global normalization was applied. Missing protein-abundance values were retained as missing and were not imputed; only proteins with non-missing values in at least two out of three replicates in at least one group were considered for statistical testing. No additional post hoc batch-correction algorithm was applied, as all samples were acquired in a single batch. For downstream differential-abundance analysis, the individual animal was considered the independent biological unit. Protein fold changes were calculated from normalized protein abundances across the three biological replicates in each group. Statistical significance was evaluated using a two-sided Student’s *t*-test (performed only on the available non-missing replicates), followed by Benjamini–Hochberg correction for multiple comparisons. Differentially abundant proteins meeting the predefined fold-change criterion and an FDR-adjusted q value < 0.05 were subsequently used for KEGG pathway enrichment analysis, cross-region intersection analysis, and metabolome–proteome co-enrichment analysis [20,21].

### 2.9. Data Processing and Metabolite Annotation

The raw data from the AFADESI-MS analysis was converted to cdf format files using Xcalibur 2.3 (Thermo Scientific, Waltham, MA, USA) and then imported into the custom-developed graphical software MassImager (Version 1.0, Beijing Viktor Technology Co., Ltd., Beijing, China) for ion image reconstruction. After background subtraction, each section was normalized to the sample total ion chromatograms (TIC) value using MassImager, and the ion images were finally presented using the TIC normalized intensity threshold. MS profiles from the microregions of the brain were precisely extracted from the MSI data, and each brain microregion profile was delineated and calculated 3 times from the subregions to reduce error. These repeated measurements were averaged to generate a single ROI mean ion intensity for each animal; they were not treated as independent biological replicates. Then, the average ion intensity of the microregion was generated separated two-dimensional data matrixes (*m*/*z*, ion intensity) in the txt format. The mass tolerance for peak pick and background subtraction was set to 0.005 Da, and the proportional coefficient k was set to 1. Thereafter, the txt file was imported into Markerview 1.2.1 (AB SCIEX, Marlborough, MA, USA) for peak alignment and isotope ion deletion. Multivariate statistical analysis was carried out using the ropls package (v1.38.0), which included principal component analysis (PCA). For differential-feature screening, comparisons between nicotine-treated and control animals were performed at the biological-animal level using a two-sided independent-sample Student’s *t*-test. *p*-values were adjusted for multiple testing using the Benjamini–Hochberg false-discovery-rate procedure, and features meeting both *p* < 0.05 and FDR-adjusted Q < 0.05 were retained for downstream differential-feature analysis. Candidate metabolite annotations were based on accurate mass, targeted high-resolution MS/MS fragmentation, and comparison with reference information in HMDB 5.0, METLIN, and MS-DIAL, with mass error generally maintained below 5 ppm. Because authentic reference standards were not available for systematic confirmation of the endogenous MSI-derived metabolites, these assignments are reported as putative annotations rather than definitive identifications. Pathway enrichment analysis was performed using the pathway analysis module of MetaboAnalyst 6.0 [22,23].

### 2.10. Statistical Analysis

Statistical analyses were performed using R version 4.3.2 and GraphPad Prism 8.0. Data are presented as mean ± SEM unless otherwise stated. For ROI-based analyses, the individual animal was considered the independent biological unit (*n* = 3 animals per group). Repeated ROI delineations or measurements within the same tissue section were treated as technical/subsampling measurements and were averaged to obtain a single ROI-level value for each animal before statistical analysis. Pairwise comparisons between the control and nicotine-treated groups were performed using a two-sided independent-sample Student’s *t*-test at the biological-animal level. For high-dimensional spatial metabolomic feature screening, the resulting *p*-values were additionally adjusted for multiple comparisons using the Benjamini–Hochberg false-discovery-rate (FDR) procedure, and differential features were defined using the prespecified criteria of *p* < 0.05 and FDR-adjusted Q < 0.05. Because of the limited biological sample size, these statistical comparisons were considered exploratory and were interpreted with appropriate caution. Principal component analysis (PCA) and related multivariate analyses were performed using the ropls package [24] to evaluate metabolic separation between the control and nicotine groups across ROIs and ionization modes. UpSet plots, bubble plots, bar plots, and feature distribution plots were generated using the R packages described above. *p* < 0.05 was considered statistically significant, with * *p* < 0.05, ** *p* < 0.01, and *** *p* < 0.001 used in the figures.

Due to the nature of the interventions, complete blinding during the entire study was not feasible. However, blinding was maintained during the critical evaluation stages.

During allocation and conduct of the experiment, the primary investigators were aware of the group allocations to ensure the correct administration of treatments and procedural accuracy.

During outcome assessment, the researchers performing the outcome assessments (e.g., mass spectrometry imaging, molecular assays, data quantification) were blinded to the treatment groups to eliminate subjective bias.

Data analysts were blinded to the group identities, working with anonymized datasets until the statistical evaluation was completed.

## 3. Results

### 3.1. A Brain–Kidney Spatial Multi-Omics Workflow Integrating In Situ Imaging and Mass Spectrometry of Laser-Microdissected Microregions

To avoid the loss of regional information caused by bulk homogenization, we established an integrated workflow encompassing animal treatment, tissue sectioning, in situ imaging, laser microdissection, targeted mass spectrometry, and proteomic analysis (Figure 1A). Optimization of the Leica LMD7 showed that 20% laser energy did not produce complete cutting boundaries, whereas 40% energy caused more pronounced edge carbonization and thermal damage. A setting of 30% produced clear boundaries with limited thermal damage in both brain and kidney tissues (Figure 1B), and was therefore used for downstream microregion collection.

Because laser-microdissected samples contain limited material, extraction reproducibility is critical for targeted quantification. Among the extraction systems tested, 80% MeOH containing 0.1% formic acid yielded more consistent recovery of nicotine and cotinine recovery than pure methanol or non-acidified mixtures (Figure 1C). This solvent system was therefore used for targeted LC-MS/MS analysis of microdissected microregions. Together, these optimizations established a workflow that preserves spatial context and enables robust downstream molecular measurements.

### 3.2. MALDI-TOF Imaging and Quantification of Laser-Microdissected Microregions Reveal Compartmentalized Brain–Kidney Distributions of Nicotine and Its Metabolites

To characterize the spatial distribution of nicotine-related molecules between the central nervous system and the kidney, a major organ of peripheral clearance, we first used MALDI-TOF MSI to visualize target ions directly in brain and kidney sections. Anatomically defined brain and kidney compartments were then isolated by laser microdissection and quantified by targeted LC-MS/MS (Figure 2), thereby combining in situ visualization with microregion-level quantitative measurement. The results showed that nicotine was detectable in both the brain and kidney after acute exposure, but levels in the renal cortex and medulla were markedly higher than those in all five brain regions, indicating pronounced renal accumulation. Within the brain, nicotine levels were relatively higher in PFC and HIPP than in OB, STR, and THA. The MALDI-TOF ion images further supported a diffuse but heterogeneous distribution in the brain and a stronger renal signal, consistent with peripheral enrichment concurrent with nicotine entry into the central nervous system.

Cotinine and nornicotine showed a similar brain–kidney distribution pattern, with substantially higher levels in the renal cortex and medulla than the brain regions, consistent with established pharmacokinetic characteristics of nicotine metabolism and urinary elimination [4,25]. By contrast, 3-hydroxycotinine showed a relatively prominent signal in OB, suggesting possible region-selective central accumulation, local conversion, or retention. Thus, nicotine and its major metabolites did not exhibit a homogeneous distribution; instead acute exposure produced a spatial pattern characterized by central entry, renal enrichment, and metabolite-specific variation. LC-MS measurements of nicotine and its metabolites in microdissected brain tissue and kidney subregions were consistent with the corresponding MSI patterns.

### 3.3. Laser-Microdissected Microregion Quantification Shows That Acute Nicotine Preferentially Amplifies Striatal Dopamine Turnover

After characterizing the spatial distribution of nicotine and its metabolites, we examined acute neurochemical responses across brain regions. Because neurotransmitter levels differ substantially across anatomical regions, OB, PFC, STR, HIPP, and THA were collected by laser microdissection and analyzed by targeted LC-MS/MS. Representative neurotransmitter distributions were visualized in parallel by MSI (Figure 3). Dopamine (DA) showed a strong baseline regional bias toward STR. After acute nicotine exposure, DA increased markedly in STR, and ion images showed enhanced signal in the striatal area (Figure 3A,B). DOPAC, a key indicator of dopamine turnover, showed a similar and more pronounced response: STR DOPAC was significantly higher in the nicotine group than in the control group, and its spatial signal was concentrated in the striatum (Figure 3C,D). The coordinated increases in DA and DOPAC are consistent with enhanced local dopamine signaling and increased post-release dopamine metabolism.

Compared with the dopamine axis, ACh, GABA, NE, and glutamate profiles were more strongly shaped by the baseline neurochemical architecture of each region. ACh was relatively abundant in STR, HIPP, and THA and tended to increase in some regions after nicotine exposure. GABA, NE, and glutamate showed more modest acute changes. These observations suggest that acute nicotine does not uniformly affect all neurotransmitter systems; instead, it preferentially engages markers of striatal dopamine turnover, whereas other transmitter systems respond in a region-dependent manner.

### 3.4. Spatial Metabolomics Reveals Regionally Heterogeneous Metabolic Remodeling Across Five Brain Regions

**Figure 4:**
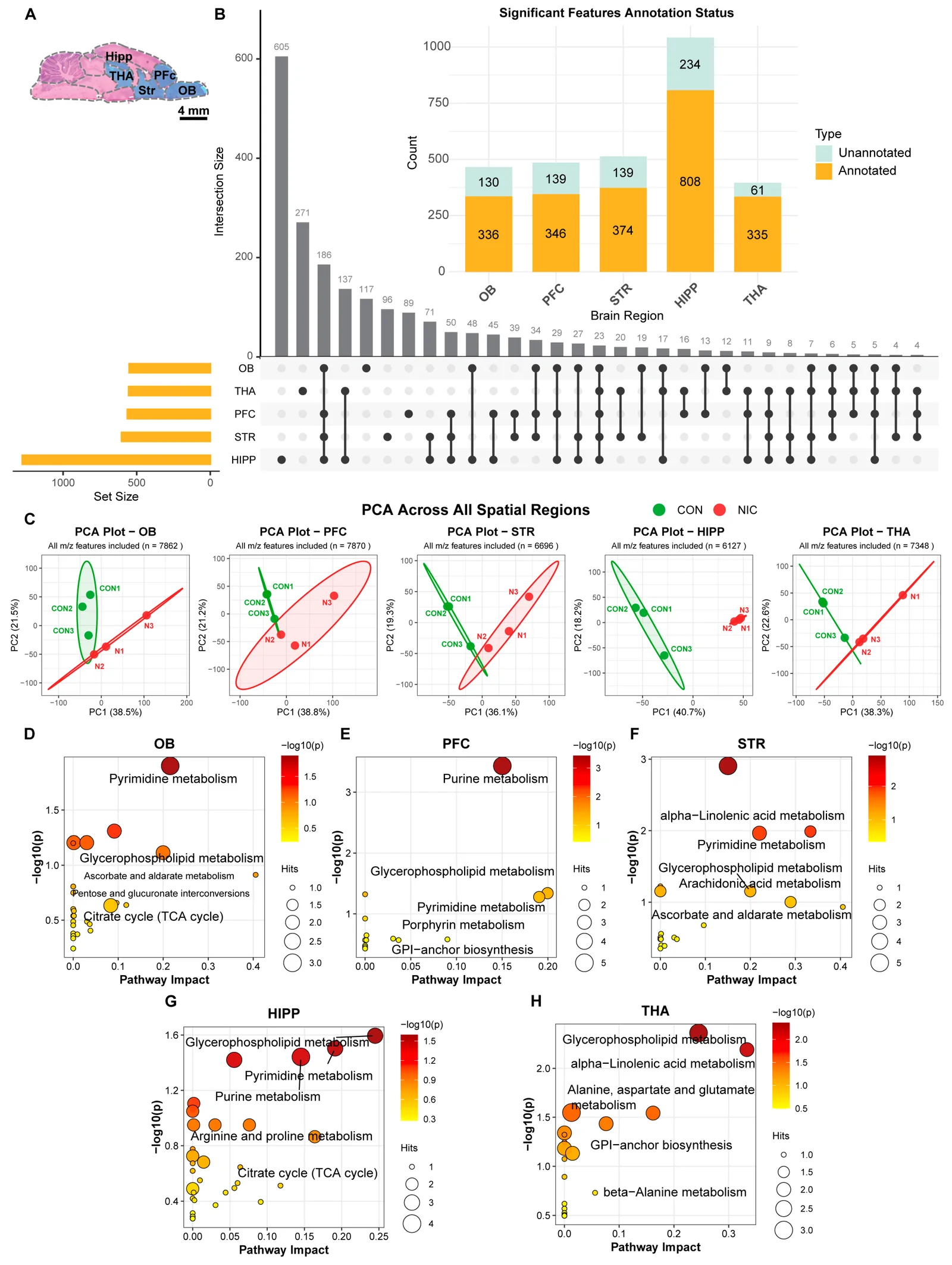
AFADESI-MSI spatial metabolomics reveals nonuniform nicotine-induced metabolic remodeling across five brain regions. (**A**) Anatomical ROI map showing the OB, PFC, STR, HIPP, and THA regions used for spatial feature extraction. (**B**) UpSet-style intersection analysis of significant metabolic features across regions, with the inset summarizing annotated and unannotated differential features in each ROI. (Note: The annotations in this section are based on MS1-level mass spectrometric information.) (**C**) PCA score plots generated from the full *m*/*z* feature matrix for each region, showing separation between the control (CON) and nicotine-treated (Nic) groups within region-specific feature spaces. (**D**–**H**) MetaboAnalyst pathway topology/enrichment bubble plots for region-specific differential metabolites in OB, PFC, STR, HIPP, and THA, respectively. The *x*-axis shows pathway impact, the *y*-axis shows −log_10_(*p* value), bubble size indicates the number of matched metabolites, and color indicates enrichment significance. The combined intersection, PCA, and pathway analyses show that acute nicotine exposure produces reproducible but region-biased metabolic remodeling, with shared involvement of nucleotide and glycerophospholipid metabolism and additional region-specific amino acid, lipid-signaling, and energy-metabolism pathways. Glycosylphosphatidylinositol (GPI).

Neurotransmitter changes provide a rapid functional readout, but they do not fully capture broader regional metabolic responses. We therefore performed AFADESI-MSI spatial metabolomics in tissue-section regions of interest (ROIs) corresponding to the five brain regions (Figure 4A). UpSet analysis showed that significant features were predominantly region-specific, with relatively limited overlap among regions (Figure 4B). These findings indicate that acute nicotine does not produce a uniform whole-brain metabolic response; instead, the response comprises multiple region-specific metabolic profiles.

HIPP contained the largest number of significant features, including 808 annotated and 234 unannotated features, indicating a broad metabolic response with many putatively annotated features. OB, PFC, STR, and THA also contained numerous annotated differential features; the annotations in this section were based on MS1-level mass spectrometric information. PCA showed clear separation between the control and nicotine groups in all five brain regions, with close within-group clustering (Figure 4C), indicating consistent group-associated metabolic differences within each region.

During data annotation and principal component analysis (PCA), we relied on MS1 information. However, for the pathway enrichment analysis of differentially expressed metabolites across brain regions between the treatment and control groups, we performed high-resolution targeted MS/MS analysis on all differential metabolite *m*/*z* values to confirm their identities. A total of 10, 14, 16, 19, and 5 differential metabolites were identified in the OB, PFC, STR, HIPP, and THA, respectively (Supporting Information S1, Table S1). These metabolites were identified by subjecting adjacent tissue sections to pretreatment, followed by targeted acquisition of high-resolution MS/MS fragment ion information at the *m*/*z* values of the differential metabolites, and comparing the spectra against the METLIN and MS-DIAL databases. KEGG enrichment analysis of these differential metabolites revealed distinct functional profiles across regions (Figure 4D–H). Specifically, OB showed enrichment of pyrimidine metabolism, glycerophospholipid metabolism, ascorbate and aldarate metabolism, and the tricarboxylic acid (TCA) cycle. PFC showed enrichment of purine, pyrimidine, and glycerophospholipid metabolism. STR showed enrichment of pyrimidine metabolism, glycerophospholipid metabolism, alpha-linolenic acid metabolism, and arachidonic acid metabolism, consistent with region-specific involvement of lipid signaling and inflammatory lipid pathways. HIPP showed broad enrichment of glycerophospholipid, purine, pyrimidine, arginine and proline metabolism, and the TCA cycle. The THA was characterized by enrichment of glycerophospholipid metabolism, alpha-linolenic acid metabolism, alanine, aspartate and glutamate metabolism, and glycosylphosphatidylinositol (GPI) anchor biosynthesis. Collectively, nucleotide and membrane lipid metabolism represented a common response axis, whereas amino acid metabolism, inflammatory lipid metabolism, and energy metabolism showed region-specific patterns.

### 3.5. MicroDIA Proteomics Reveals Region-Specific Protein Remodeling After Acute Nicotine Exposure

**Figure 5:**
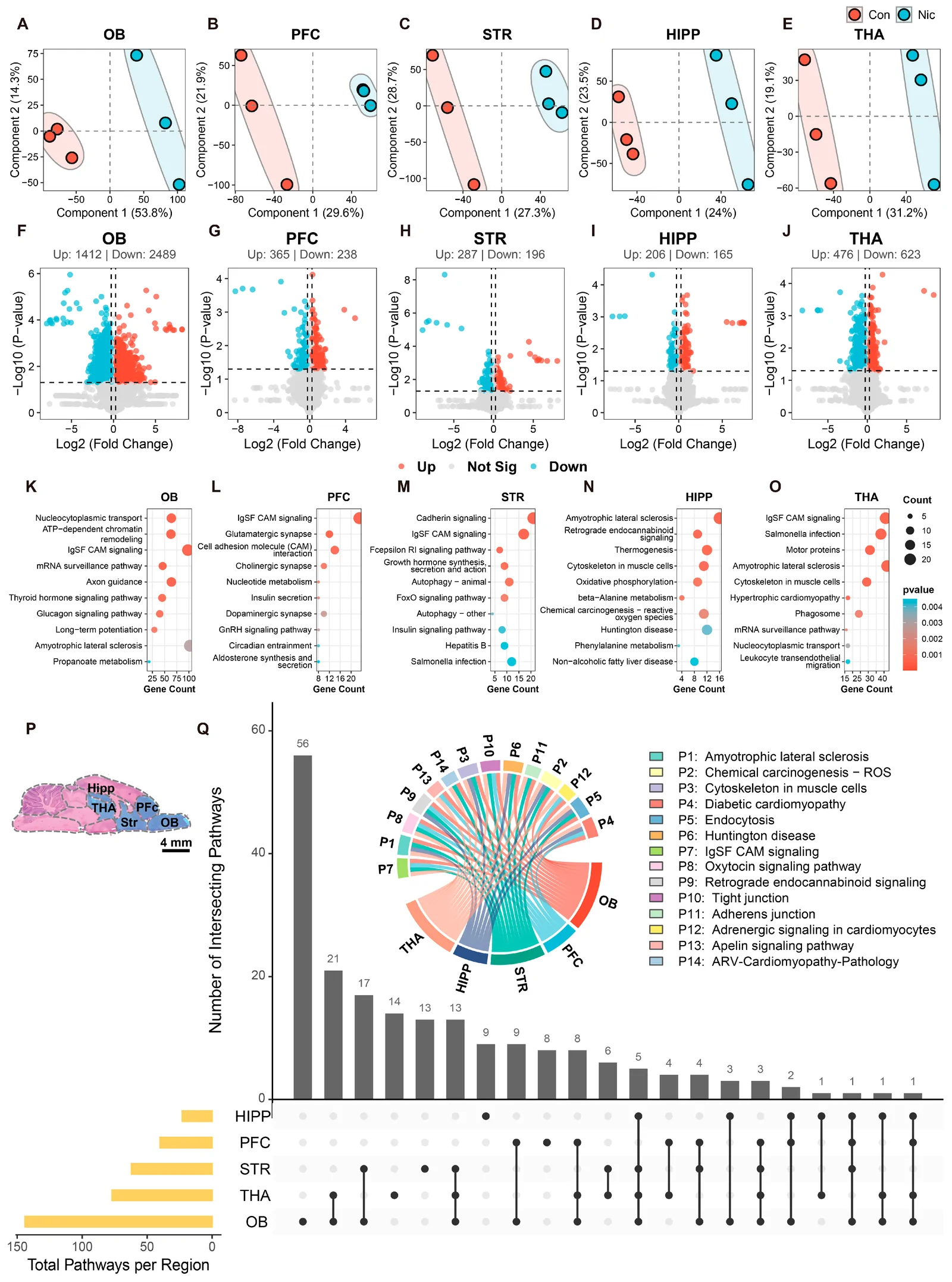
Proteomics of laser-microdissected microregions identifies brain-region-specific protein remodeling after acute nicotine exposure. (**A**–**E**) Dimensionality-reduction plots of microDIA proteomes from LMD-collected OB, PFC, STR, HIPP, and THA regions, showing separation between control (Con) and nicotine-treated (Nic) samples within each anatomical compartment. (**F**–**J**) Volcano plots of differential proteins in the same regions. Red and blue points denote significantly upregulated and downregulated proteins, respectively, and gray points denote proteins not passing the differential threshold; the *x*-axis indicates log_2_(fold change), and the *y*-axis indicates −log_10_(*p* value). The overall screening criteria for the volcano plot were: log_2_(Fold Change) > 0.5 and *p* < 0.05. (**K**–**O**) KEGG pathway enrichment of differential proteins, highlighting region-dependent changes in transport, cell adhesion/cytoskeletal organization, synaptic signaling, nucleotide/energy metabolism, autophagy, oxidative stress, and immune-related pathways. (**P**) Anatomical map of the five LMD brain ROIs. (**Q**) Pathway-intersection analysis summarizing shared and region-specific proteomic response modules across ROIs. All panels are based on anatomically isolated microregions rather than bulk brain homogenates, allowing local protein changes to be matched to the spatial framework used for targeted quantification and metabolomics.

Because metabolic remodeling alone does not define the associated protein pathways, we next analyzed laser-microdissected OB, PFC, STR, HIPP, and THA by microDIA proteomics. Each proteomic sample was derived from an anatomically defined microregion, enabling a more direct interpretation of local protein expression than would be possible using mixed tissue homogenates. Dimensionality reduction analyses separated control and nicotine samples within each region (Figure 5A–E), indicating region-specific proteomic differences associated with acute nicotine exposure. The numbers of differential proteins varied markedly by region: OB had 1412 upregulated and 2489 downregulated proteins, representing the largest differential set; THA had 476 upregulated and 623 downregulated proteins; PFC, STR, and HIPP showed smaller but distinct proteomic responses (Figure 5F–J). Thus, the proteomic effects of acute nicotine differed among brain regions, with the largest numbers of differential proteins observed in OB and THA.

KEGG enrichment analysis identified distinct functional profiles across regions (Figure 5K–O). OB showed enrichment of nucleocytoplasmic transport, ATP-dependent chromatin remodeling, IgSF CAM signaling, mRNA surveillance, and axon guidance, suggesting active cellular program regulation and neural structural remodeling. PFC enrichment involved cell adhesion, glutamatergic synapse, cholinergic synapse, nucleotide metabolism, and dopaminergic synapse pathways, reflecting coupling between synaptic communication and metabolic regulation. STR was enriched for cadherin signaling, IgSF CAM signaling, FoxO signaling, autophagy, and insulin signaling. HIPP showed enrichment of retrograde endocannabinoid signaling, oxidative phosphorylation, thermogenesis, and ROS-related pathways, pointing to energy and oxidative-stress regulation. THA was enriched for IgSF CAM signaling, motor proteins, cytoskeleton, phagosome, and nucleocytoplasmic transport, indicating structural remodeling, transport homeostasis, and immune-related processes. Pathway intersection analysis further showed limited sharing across regions, with region-specific pathways dominating (Figure 5P,Q). Representative shared pathways included IgSF CAM signaling, endocytosis, retrograde endocannabinoid signaling, tight junction, adherens junction, and oxidative-stress/neurodegeneration-related pathways. Overall, the proteomic response combined recurrent stress- and membrane-transport-associated pathways with pronounced regional specificity.

### 3.6. Metabolome–Proteome Co-Enrichment Identifies Shared Pathways and Region-Specific Coupling

**Figure 6:**
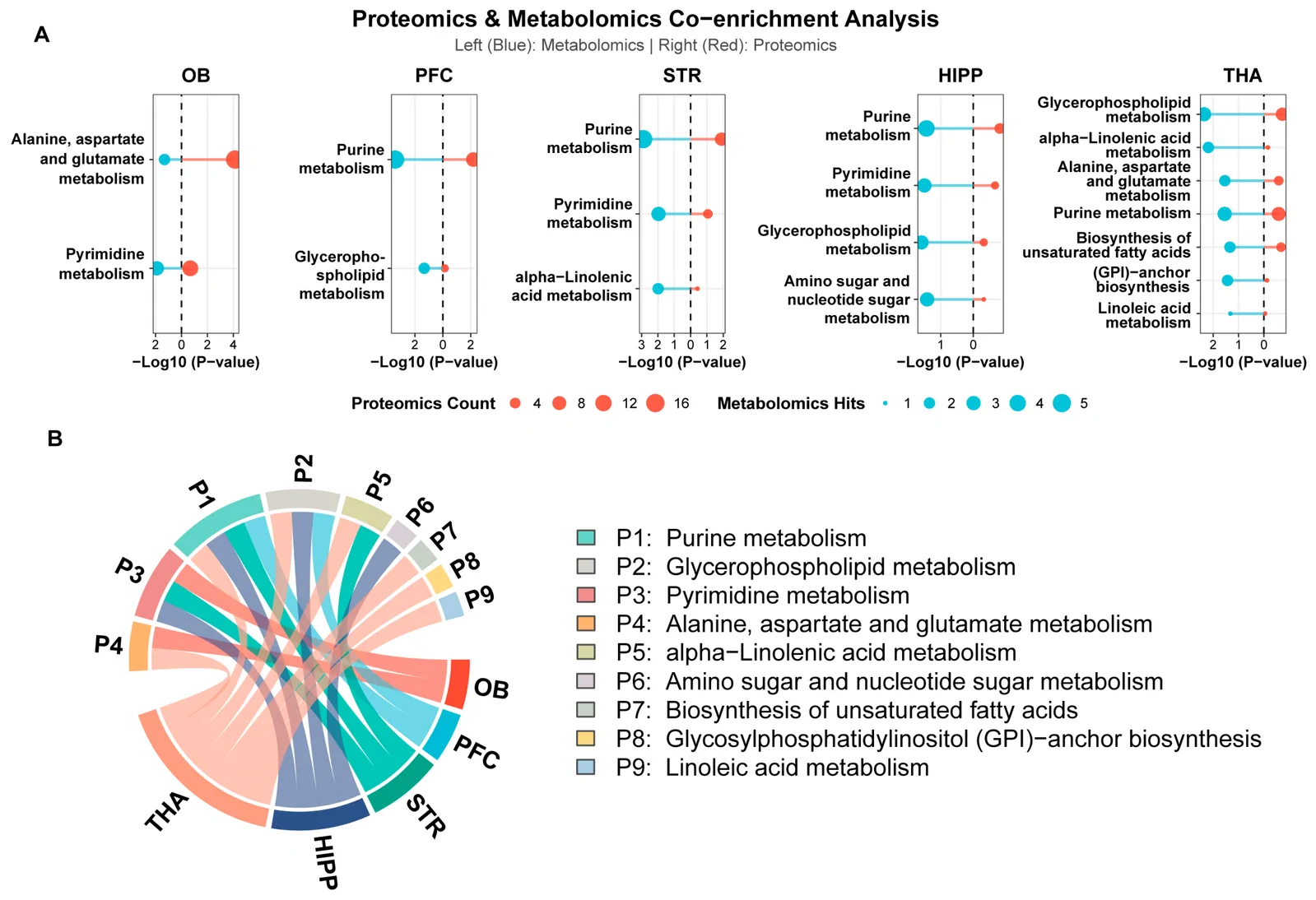
Cross-omics co-enrichment links spatial metabolites and microregion proteins to shared functional pathways. (**A**) Paired bubble plots showing pathways co-enriched in AFADESI-MSI metabolomics (left/blue side of each panel) and LMD-microDIA proteomics (right/red side) for OB, PFC, STR, HIPP, and THA. The horizontal position encodes −log_10_(*p* value), and bubble size denotes the number of metabolite hits or proteins contributing to each pathway. (**B**) Brain region–pathway chord diagram summarizing the distribution of co-enriched pathways across anatomical regions. Pathway codes: P1, purine metabolism; P2, glycerophospholipid metabolism; P3, pyrimidine metabolism; P4, alanine, aspartate and glutamate metabolism; P5, alpha-linolenic acid metabolism; P6, amino sugar and nucleotide sugar metabolism; P7, biosynthesis of unsaturated fatty acids; P8, glycosylphosphatidylinositol (GPI)-anchor biosynthesis; and P9, linoleic acid metabolism. The analysis indicates that nucleotide and membrane-lipid pathways form recurrent cross-omics axes, while THA displays the broadest metabolite–protein coordination and other regions show more selective pathway coupling.

To assess whether the metabolomic and proteomic layers converged on common functional modules, we performed co-enrichment analysis of AFADESI spatial metabolomics and microDIA proteomics from laser-microdissected microregions (Figure 6A). Proteomic data for each brain region are provided in Supporting Information S2 (SI2). The five brain regions did not share an identical cross-omics response. Instead, a small number of common pathways were superimposed on strong regional specificity. Purine metabolism appeared in PFC, STR, HIPP, and THA, suggesting that nucleotide turnover and energy-related remodeling form a common foundation. Pyrimidine metabolism appeared in OB, STR, and HIPP, indicating changes in nucleic-acid precursor metabolism and biosynthetic demand. Glycerophospholipid metabolism was concentrated in PFC, HIPP, and THA, suggesting region-preferential membrane lipid remodeling.

Region-level coupling was highly informative. OB mainly showed co-enrichment of alanine/aspartate/glutamate metabolism and pyrimidine metabolism. PFC was concentrated around purine and glycerophospholipid metabolism. STR involved purine, pyrimidine, and alpha-linolenic acid metabolism, consistent with its neurotransmitter and lipid-signaling responses. HIPP covered purine, pyrimidine, glycerophospholipid, and amino sugar/nucleotide sugar metabolism. THA showed the broadest co-enrichment spectrum, including purine metabolism, glycerophospholipid metabolism, alpha-linolenic acid metabolism, alanine/aspartate/glutamate metabolism, unsaturated fatty acid biosynthesis, GPI-anchor biosynthesis, and linoleic acid metabolism (Figure 6B). These findings suggest particularly broad acute nicotine-associated metabolite–protein coordination in the thalamus.

### 3.7. Microregional Quantitation and Imaging of Key Bioenergetic and Neuromodulatory Signatures

**Figure 7:**
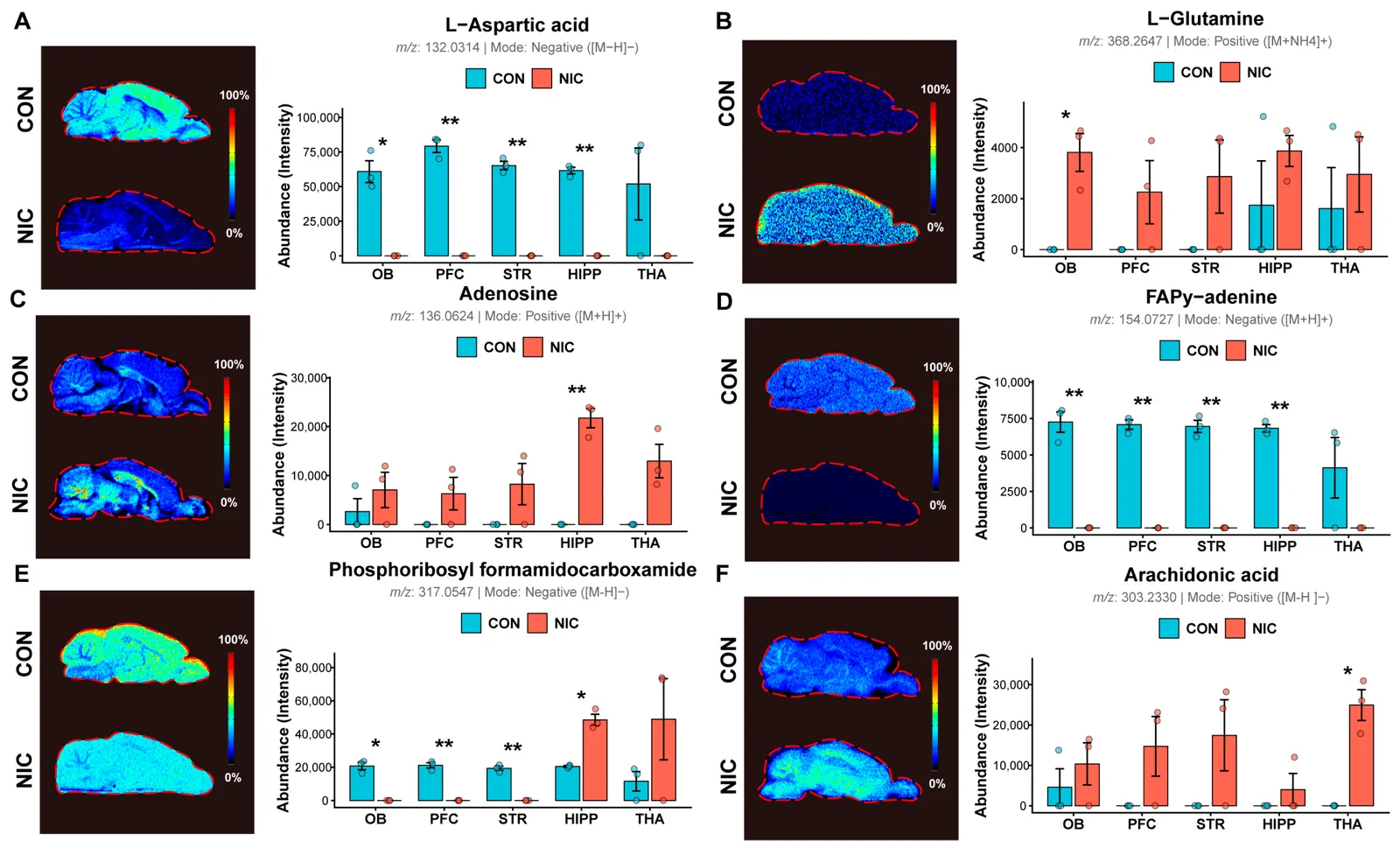
Spatial imaging and ROI quantification of representative candidate metabolites in response to nicotine. (**A**–**F**) AFADESI-MSI ion images and region-wise ROI intensity bar plots for selected features assigned to (**A**), L-aspartic acid, (**B**), L-glutamine, (**C**), adenosine, (**D**), FAPy-adenine, (**E**), phosphoribosyl formamidocarboxamide (FAICAR), and (**F**), arachidonic acid. For each feature, the images on the left compare spatial distribution in control (CON) and nicotine-treated (NIC) brain sections, whereas the plots on the right summarize normalized mean ROI mean ion intensities in the OB, PFC, STR, HIPP, and THA. Heat-map colors indicate normalized ion abundance from low to high (0–100%), and overlaid points on the bar plots represent individual ROI measurements. These features were selected to represent pathways implicated by cross-omics enrichment, mainly purine/pyrimidine metabolism, urate-related purine catabolism, and amino acid metabolism. Several assignments are database-level candidate annotations based on accurate mass and spatial plausibility. * *p* < 0.05, ** *p* < 0.01.

To relate these co-enriched pathways to specific spatial profiles, we quantified regional changes in the abundance of six key intermediate metabolites in the OB, PFC, STR, HIPP, and THA under control (CON) and nicotine-challenged (NIC) conditions (Figure 7).

Consistent with the co-enriched alanine, aspartate, and glutamate metabolism pathway, an acute nicotine challenge induced a substantial reciprocal remodeling of key neurotransmitter precursors. L-Aspartic acid (Figure 7A) was heavily and significantly depleted in the NIC group across all analyzed brain regions compared to the CON group, falling to near-baseline levels in the OB, PFC, STR, and HIPP. Conversely, L-glutamine (Figure 7B) increased across regions after nicotine exposure. The increase was most pronounced and statistically significant in the OB, with upward trends in the PFC, STR, HIPP, and THA; this pattern is consistent with altered astrocytic–neuronal glutamate–glutamine–aspartate cycling. Adenosine (Figure 7C) exhibited a robust, global elevation in the NIC group across all five brain microregions. This neuromodulatory surge peaked dramatically and with high statistical significance in the HIPP, while demonstrating massive upward trends in the OB, PFC, STR, and THA, signaling a widespread bioenergetic feedback response. Crucially, FAPy-adenine (formamidopyrimidine-adenine; Figure 7D), an oxidized purine base intermediate, was completely ablated across all five brain regions in the NIC group, dropping from high baseline levels in the CON group to near-undetectable limits. This points to an absolute suppression of standard purine degradation or oxidative lesion accumulation during acute exposure. In stark contrast to the uniform trends above, the de novo purine biosynthetic intermediate phosphoribosyl formamidocarboxamide (FAICAR; Figure 7E) exhibited a highly region-specific, divergent allocation dichotomy. In the anterior and reward-related hubs, the OB, PFC, and STR, FAICAR levels were significantly down-regulated in the NIC group. Conversely, FAICAR heavily accumulated in the posterior and deeper macro-networks, showing a significant surge in the HIPP and a prominent upward trend in the THA. Arachidonic acid (Figure 7F) levels were significantly elevated in all five brain regions examined.

Because several metabolites shown in Figure 7 are database-level candidate annotations, their individual molecular identities should be interpreted cautiously; we therefore emphasize pathway-level findings and spatial patterns. Collectively, these representative features are consistent with broad changes in nucleotide, amino acid, and oxidative metabolic networks after acute nicotine exposure.

## 4. Discussion

The principal contribution of this study is its characterization of acute nicotine exposure as a spatially organized brain–kidney molecular response rather than an exclusively central neuropharmacological response. By integrating MALDI-TOF MSI, AFADESI spatial metabolomics, laser microdissection-based targeted quantification, and microDIA proteomics, we examined nicotine-related molecular distributions, brain-region-specific neurotransmitter changes, endogenous metabolic remodeling, and protein pathway regulation within a common anatomical framework. This spatial approach can capture compartmental organization that may be obscured by bulk homogenization. Nicotine was detected in multiple brain regions and was accompanied by neurochemical responses, whereas nicotine, cotinine, and nornicotine were much more strongly enriched in the renal cortex and medulla. This distribution is consistent with rapid nicotine metabolism and urinary elimination [4]. These findings suggest that the central effects of acute nicotine cannot be inferred from brain nicotine abundance alone; peripheral clearance rate, renal compartmental transport, and metabolite pool size may influence the timing and duration of central exposure. Moreover, local nicotine abundance did not fully correspond to the magnitude of the regional molecular response. The PFC and HIPP showed relatively high nicotine signals, yet the strongest neurotransmitter response involved dopamine and DOPAC in the STR, whereas the strongest proteomic remodeling occurred in the OB and THA. This discordance suggests that regional receptor profiles, circuit architecture, cell composition, energy state, and metabolic buffering capacity may contribute to the direction and magnitude of downstream responses after nicotine enters the brain. In particular, the rapid increase in striatal dopamine turnover provides a direct link to established addiction neurobiology. Nicotine modulates dopaminergic neuronal excitability and terminal release through nicotinic acetylcholine receptor-dependent mechanisms [2,3,26,27,28]. The concurrent increases in STR DA and DOPAC are consistent with enhanced dopamine signaling and accelerated post-release metabolism. By contrast, ACh, GABA, NE, and glutamate varied more strongly according to regional baseline context, suggesting that acute nicotine preferentially engages the striatal dopamine axis while also acting within local neurotransmitter systems.

Cross-omics analyses indicated that acute nicotine was associated with changes in broader metabolic and proteomic networks in addition to individual neurotransmitters. Co-enrichment of purine and pyrimidine pathways implicates processes related to ATP turnover, nucleotide salvage, RNA-associated stress, and early cellular adaptation [29]. Enrichment of glycerophospholipid, alpha-linolenic acid, and linoleic acid pathways suggests the involvement of membrane organization, synaptic vesicle dynamics, receptor localization, and inflammatory lipid signaling. Proteomic enrichment of cell adhesion, endocytosis, tight junction, adherens junction, and retrograde endocannabinoid signaling provides complementary evidence linking membrane lipid remodeling to functional network reorganization. The regional specificity of these patterns may be biologically relevant. The OB showed the strongest proteomic perturbation, suggesting particular sensitivity to nicotine-associated cellular remodeling. The HIPP contributed the largest number of spatial metabolic features, consistent with its the sensitivity of hippocampal processes to energy balance, synaptic plasticity, and oxidative stress. The THA showed the broadest metabolite–protein co-enrichment, encompassing purine metabolism, glycerophospholipid metabolism, fatty acid metabolism, unsaturated fatty acid biosynthesis, GPI-anchor biosynthesis, and amino acid metabolism. Given the role of the thalamus in sensory, motor, arousal, and limbic integration, this broad response may be relevant to the wider functional consequences of acute nicotine exposure. Overall, this study suggests the unique value of integrating microdissection-based spatial proteomics and mass spectrometry imaging-based spatial metabolomics in investigating the mechanisms of drug action on brain tissue [30].

By avoiding bulk tissue homogenization, our spatial cross-omics framework suggests that acute nicotine exposure triggers strictly compartmentalized, region-specific molecular execution programs rather than a uniform central surge. Nicotinic hyper-activation via α4\β2 and α7 nicotinic acetylcholine receptors (nAChRs) demands immense adenosine triphosphate (ATP) consumption, provoking a significant localized elevation of extracellular adenosine, especially in the hippocampus, that acts as a retrograde inhibitory neuromodulator to suppress excitotoxic dopamine and glutamate over-release [31,32]. This bioenergetic crisis likely induces a purine-sparing response in the central nervous system, consistent with the marked reduction of FAPy-adenine across all microregions. This observation may point to a suppression of standard purine degradation [33] and a compensatory reliance on salvage pathways, though these interpretations remain tentative given the limited sample size [33]. The divergent regional pattern of the de novo purine intermediate FAICAR, which decreased in the OB, PFC, and STR, increased in the HIPP, and showed an upward trend in the THA, may reflect regional differences in purine biosynthetic demand or regulation [34]. Concurrently, in the nAChR-dense sensory and gating networks of the OB and THA, the absolute depletion of L-aspartic acid paired with a universal surge in L-glutamine is suggestive of a hypothesized astrocytic clearance response—namely, an accelerated glutamate–glutamine–aspartate shuttle mobilized to clear hyper-synaptic glutamate during nicotinic firing and prevent neurotoxicity. However, this interpretation remains speculative and requires functional validation through cell-type-specific flux studies [35,36,37]. Coupled with immediate membrane structural adaptations via synchronized glycerophospholipid and α-linolenic acid metabolism to support rapid vesicle recycling and receptor endocytosis [38], these cross-omics signatures suggest acute nicotine challenge as a highly localized, hierarchical process. Furthermore, several limitations should be noted. First, some representative metabolites in Figure 7 are database-level candidate annotations and require confirmation using standards. Second, this study focuses on early molecular responses after acute high-dose challenge and does not address chronic exposure, withdrawal, dose gradients, sex differences, or long-term behavioral outcomes. The acute challenge dose used in this study (15 mg/kg i.p., following a 3-day low-dose adaptation) represents a high-dose pharmacological/toxicological challenge. While this high dose was selected to ensure robust molecular mobilization and signal intensity across microdissected microregions, it approaches sub-convulsive thresholds and inevitably triggers substantial autonomic, endocrine, and systemic stress responses. Consequently, molecular shifts such as global adenosine accumulation, arachidonic acid surge, and aspartate depletion likely reflect, in part, non-specific acute bioenergetic stress and neuroendocrine activation. Caution must be exercised when extrapolating these findings to low-dose, behaviorally rewarding, or chronic voluntary exposure scenarios. Third, while our cross-omics integration successfully mapped shared functional modules through pathway-level co-enrichment, direct quantitative correlation modeling (such as enzyme–metabolite pairwise correlation networks or multi-block sparse PLS) was not performed in this foundational study. Given the high dimensionality of microregion proteomes and spatial metabolomes, quantitative correlation modeling across static acute exposure states carries a risk of overfitting. Future studies incorporating expanded dose gradients, temporal kinetic tracking, and targeted omics will be essential to quantitatively decipher direct enzyme–metabolite regulatory dynamics within these localized compartments. Finally, while our spatial multi-omics framework leveraged laser microdissection to significantly attenuate tissue heterogeneity variance, achieving distinct group separation in multivariate analyses (PCA), the biological sample size (*n* = 3 per group for microregion omics and LMD quantification) remains relatively modest. Although an *n* = 3 structure is standard in labor- and cost-intensive spatial omics research, future studies featuring expanded animal cohorts and dose–response gradients will be valuable to further confirm subtle microregional alterations.

## 5. Conclusions

In conclusion, this study presents a spatial multi-omics framework that offers a spatially resolved view of acute nicotine exposure as a compartmentalized rather than homogeneous systemic event, involving peripheral renal handling, region-dependent central distribution, and localized molecular responses. By integrating in situ mass spectrometry imaging with laser-microdissected microregion proteomics and targeted quantification, this approach reduced the limitations associated with conventional bulk tissue homogenization and linked pharmacokinetic disposition with functional neurochemical and proteomic phenotypes.

Our findings showed marked central–peripheral differences, with nicotine and its primary metabolites enriched within the renal cortex and medulla and nicotine distributed heterogeneously across brain regions. Within the central nervous system, acute nicotine was associated with concurrent increases in striatal dopamine and DOPAC consistent with enhanced dopamine turnover, as well as nonuniform, region-specific metabolic and proteomic changes across distinct brain microregions. Cross-omics integration identified nucleotide and membrane lipid metabolism as recurrent response pathways, with the THA showing the broadest metabolite–protein co-enrichment. The global increase in adenosine, near-undetectable signal assigned to FAPy-adenine, and opposing regional changes in FAICAR suggest region-dependent alterations in purine metabolism and related bioenergetic and neuromodulatory processes, but do not establish specific mechanisms. Likewise, the decrease in L-aspartic acid and regionally varying increases in L-glutamine are consistent with altered amino acid metabolism and glutamate-related cycling rather than direct evidence of accelerated astrocytic clearance or protection from excitotoxicity. Collectively, these spatially resolved findings provide a foundation for further investigation of microregional neuropharmacology, bioenergetics, and membrane-associated changes during acute nicotine exposure.

## Data Availability

The original contributions presented in this study are included in the article/Supplementary Materials. Further inquiries can be directed to the corresponding authors.

## Supplementary Materials

The following supporting information can be downloaded at: https://www.mdpi.com/article/10.3390/metabo16090657/s1, Table S1: Discriminating metabolites based on AFADESI-MSI data between the treatment groups and control groups; Table S2.1. Gradient elution conditions for liquid chromatography; Table S2.2. MRM parameters for the target analytes and internal standards; Table S2.3. Linear ranges, limits of detection, and limits of quantification of the compounds; Table S2.4. Intra-day and inter-day precision and accuracy of the method; Table S2.5. Matrix effect, extraction recovery and stability of the method; Table S3.1. Gradient elution conditions for liquid chromatography; Table S3.2. MRM parameters for the target analytes and internal standards; Table S3.3. Linear ranges, limits of detection, and limits of quantification of the neurotransmitters; Table S3.4. Intra-day and inter-day precision and accuracy of the method; Table S3.5. Matrix effect, extraction recovery and stability of the method.
