# Supplementary material for: Spatially Resolved Multi-Omics Reveals Brain–Kidney Compartmentalization and Region-Specific Molecular Reprogramming After Acute Nicotine Exposure
Source: Metabolites. 2026 Sep 8;16(9):657. doi: 10.3390/metabo16090657 (PMC13609564; doi:10.3390/metabo16090657)
Supplement: Supplementary file 1 [file metabolites-16-00657-s001.zip › Supporting information S1_SI1.pdf]

Table S1. Discriminating metabolites based on AFADESI-MSI data between the treatment groups and control groups.

Optimal_Name	Formula	Measured m/z	Theoretical m/z	Ion type	Product ions	ppm	P-value	FDR-corrected Q-value	Brain region
7-Methylguanine	C6H7N5O	166.0782	166.0775	[M+H]+	149.0509;135.0534;119.0591	4.45	0.0045	0.0133	OB
Oxalacetic acid	C4H4O5	132.9987	132.9985	[M-H]-	87.0079;58.9886;	1.15	0.0059	0.0133	OB
Ascorbic acid	C6H8O6	175.0244	175.0242	[M-H]-	115.0036;86.9847;58.9891	0.96	0.0059	0.0133	OB
S-Nitrosoglutathione(GSNO)	C10H16N4O7S	337.0825	334.0823	[M-H]-	307.0570;160.9888,;130.9979	0.73	0.0091	0.0168	OB
Phosphoribosyl formamidocarboxamide	C10H15N4O9P	317.0579	317.0574	[M-H]-	298.0540;170.1461;140.0463	1.54	0.0115	0.0185	OB
L-Aspartic acid	C4H7NO4	132.0319	132.0314	[M-H]-	114.9197;73.8922;55.8816	3.85	0.0161	0.0218	OB
FAPy-adenine	C5H7N5O	154.0733	154.0727	[M+H]+	137.0461;119.0352;92.1495	4.07	0.0310	0.0315	OB
Uridine 2',3'-cyclic phosphate	C9H11N2O8P	305.0168	305.0158	[M-H]-	114.9067;96.9659;211.0120;78.9591	3.3	0.0066	0.0134	OB
dUMP	C9H13N2O8P	306.0300	306.0295	[M-H]-	242.0109;174.9891;111.9939	1.76	0.0235	0.0281	OB

LPC(16:0)	C24H49NO7P	544.3238	544.3238	[M−H]-	184.0743;104.1081				
dUMP	C9H13N2O8P	306.0300	306.0295	[M-H]-	242.0109;174.9891;111.9939	1.76	0.0235	0.0281	PFC
Adenine	C5H5N5	136.0628	136.0624	[M+H]+	119.0353;92.0495	2.93	0.0030	0.0133	PFC
Protoporphyrin IX	C34H34N4O4	563.2680	563.2656	[M+H]+	508.2576;392.2458	4.18	0.0009	0.0133	PFC
Aspartyl-Histidine	C10H14N4O5	302.1110	302.1098	[M+H]+	133.0983;110.0715;166.0872	3.9	0.0027	0.0133	PFC
Phosphoglycolic acid	C2H5O6P	155.9677	155.9669	[M-H]-	111.9775;95.9831	4.86	0.0030	0.0133	PFC
L-Aspartic acid	C4H7NO4	132.0319	132.0314	[M-H]-	114.9197;73.8922;55.8816	3.85	0.0161	0.0218	PFC
Ascorbic acid	C6H8O6	175.0244	175.0242	[M-H]-	115.0036;86.9847;58.9891	0.96	0.0059	0.0133	PFC
Phosphoribosyl formamidocarboxamide	C10H15N4O9P	317.0579	317.0574	[M-H]-	298.0540;170.1461;140.0463	1.54	0.0115	0.0185	PFC
Uridine 2',3'-cyclic phosphate	C9H11N2O8P	305.0168	305.0158	[M-H]-	114.9067;96.9659;211.0120;97.0060;78.9591	3.3	0.0066	0.0134	PFC
3-Oxalomalic acid	C6H6O7	189.0035	189.0032	[M-H]-	145.0134;127.0025;99.0079	1.74	0.0086	0.0164	PFC

7-Methylguanine	C6H7N5O	166.0782	166.0775	[M+H]+	149.0509;135.0534;119.0591	4.45	0.0045	0.0133	PFC
FAPy-adenine	C5H7N5O	154.0733	154.0727	[M+H]+	137.0461;119.0352;92.1495	4.07	0.0310	0.0315	PFC
PI(38:4)	C45H77O13P	865.5107	865.5067	[M-H]-	241.0115;223.0004	4.71	0.0021	0.0133	PFC
Phenylalanylglutamic acid	C14H18N2O5	295.1299	295.1294	[M+H]+	120.0807;84.0446;130.0491	1.59	0.0302	0.0315	PFC
Linolenic acid	C18H30O2	277.2184	277.2173	[M-H]-	195.1376;177.1281;135.0815	4.17	0.0059	0.0133	STR
Arachidonic acid	C20H32O2	303.2331	303.233	[M-H]-	179.1071;161.0958;135.1179	3.48	0.0059	0.0133	STR
L-Aspartic acid	C4H7NO4	132.0318	132.0314	[M-H]-	88.0393;74.0242;71.0139;58.0293	2.85	0.0021	0.0133	STR
Phosphoglycolic acid	C2H5O6P	155.9677	155.9669	[M-H]-	111.9775;95.9831	4.86	0.0030	0.0133	STR
Phosphoribosyl formamidocarboxamide	C10H15N4O9P	317.0579	317.0574	[M-H]-	298.0540;170.1461;140.0463	1.54	0.0115	0.0185	STR
dipamine	C8H11NO2	152.0480	152.0477	[M+H]+	137.0597;119.1491	1.97	0.0001	0.0003	STR
3,4-Dihydroxyphenylacetic acid	C8H8O4	169.0488	169.0495	[M+H]+	151.0395;123.0446	4.14	0.0039	0.0135	STR

7-Methylguanine	C6H7N5O	166.0782	166.0775	[M+H]+	149.0509;135.0534;119.0591	4.45	0.0045	0.0133	STR
Aspartyl-Histidine	C10H14N4O5	302.1110	302.1098	[M+H]+	133.0983;110.0715;166.0872	3.9	0.0027	0.0133	STR
Ascorbic acid	C6H8O6	175.0244	175.0242	[M-H]-	115.0036;86.9847;58.9891	0.96	0.0059	0.0133	STR
Uridine 2',3'-cyclic phosphate	C9H11N2O8P	305.0168	305.0158	[M-H]-	114.9067;96.9659;211.0120;97.0060;78.9591	3.3	0.0066	0.0134	STR
FAPy-adenine	C5H7N5O	154.0733	154.0727	[M+H]+	137.0461;119.0352;92.1495	4.07	0.0310	0.0315	STR
Uridine triphosphate	C9H15N2O15P3	482.9613	482.9606	[M-H]-	306.0295;158.9783;112.9865	1.35	0.0118	0.0185	STR
Noradrenochrome	C8H8NO3	164.0352	164.0350	[M-H]-	146.0244;118.0295;90.0346	0.14	0.0141	0.0210	STR
Phenylalanylglutamic acid	C14H18N2O5	295.1299	295.1294	[M+H]+	120.0807;84.0446;130.0491	1.59	0.0302	0.0315	STR
Threonylthreonine	C8H16N2O5	221.1132	221.1124	[M+H]+	221.1124;193.1175;185.1363	3.61	0.0178	0.0236	STR
dUMP	C9H13N2O8P	306.0300	306.0295	[M-H]-	242.0109;174.9891;111.9939	1.76	0.0235	0.0281	STR
Lysophosphatidylcholine(18:1)	C26H52NOP	572.3547	572.3551	[M-H]-	104.11069	2.71	0.0087	0.0134	STR

L-Arginine	C6H14N4O2	175.1197	175.119	[M+H]+	116.0715;760.0565	3.41	0.008	0.0160	HIPP
Phosphoglycolic acid	C2H5O6P	155.9677	155.9669	[M-H]-	111.9775;95.9831	4.86	0.0030	0.0133	HIPP
Oxalacetic acid	C4H4O5	132.9987	132.9985	[M-H]-	87.0079, 58.9886, 44.9982	1.15	0.0059	0.0133	HIPP
S-Nitroso-L-cysteinylglycine	C5H9N3O4S	206.0256	206.0248	[M-H]-	177.9992;130.9982;74.9602	3.98	0.0004	0.0133	HIPP
L-Aspartic acid	C4H7NO4	132.0319	132.0314	[M-H]-	114.9197;73.8922;55.8816	3.85	0.0161	0.0218	HIPP
Ascorbic acid	C6H8O6	175.0244	175.0242	[M-H]-	115.0036;86.9847;58.9891	0.96	0.0059	0.0133	HIPP
acetylcholine	C7H16NO2	146.1168	146.1176	[M]+	104.1067;87.0801;60.0808	4.82	0.003	0.0031	HIPP
Phenylalanylglutamic acid	C14H18N2O5	295.1299	295.1294	[M+H]+	120.0807;84.0446;130.0491	1.59	0.0302	0.0315	HIPP
Valyl-Arginine	C11H23N5O3	298.1832	298.1828	[M+H]+	72.0813;175.1190;129.1022	1.22	0.0034	0.0133	HIPP
Aspartyl-Histidine	C10H14N4O5	302.1110	302.1098	[M+H]+	133.0983;110.0715;166.0872	3.9	0.0027	0.0133	HIPP
L-Lysine	C6H15ClN2O2	147.1130	147.1128	[M+H]+	130.0862;84.0808;56.0498	1.27	0.0057	0.0133	HIPP
dUMP	C9H13N2O8P	306.0300	306.0295	[M-H]-	242.0109;174.9891;111.9939	1.76	0.0235	0.0281	HIPP
FAPy-adenine	C5H7N5O	154.0733	154.0727	[M+H]+	137.0461;119.0352;92.1495	4.07	0.0310	0.0315	HIPP

3-Oxalomalic acid	C6H6O7	189.0035	189.0032	[M-H]-	145.0134;127.0025;99.0079	1.74	0.0086	0.0164	HIPP
Phosphoribosyl formamidocarboxamide	C10H15N4O9P	317.0579	317.0574	[M-H]-	298.0540;170.1461;140.0463	1.54	0.0115	0.0185	HIPP
Diguanosine diphosphate	C20H26N10O15P2	697.1048	697.1028	[M-H]-	361.0432;152.0463;134.0357	2.91	0.0134	0.0204	HIPP
Homovanillic acid sulfate	C9H10O7S	261.0027	261.0024	[M-H]-	165.0556;137.0603;119.0498	1.09	0.0209	0.0271	HIPP
Glycyl-Histidine	C8H12N4O3	213.0992	213.0987	[M+H]+	181.0860;137.0961;119.0855	2.57	0.0310	0.0315	HIPP
Uridine triphosphate	C9H15N2O15P3	482.9613	482.9606	[M-H]-	306.0295;158.9783;112.9865	1.35	0.0118	0.0185	HIPP
Uridine diphosphate glucose	C15H24N2O17P2	565.0558	565.0532	[M-H]-	323.0275;241.9812;79.9661	4.66	0.0446	0.0446	HIPP
Valyl-Arginine	C11H23N5O3	298.1832	298.1828	[M+H]+	72.0813;175.1190;129.1022	1.22	0.0034	0.0133	THA
L-Aspartic acid	C4H7NO4	132.0319	132.0314	[M-H]-	114.9197;73.8922;55.8816	3.85	0.0161	0.0218	THA
Ascorbic acid	C6H8O6	175.0244	175.0242	[M-H]-	115.0036;86.9847;58.9891	0.96	0.0059	0.0133	THA

Malic acid	C4H6O5	133.0150	133.0144	[M-H]-	115.0038;89.0245	4.71	0.0302	0.0315	THA
Linolenic acid	C18H30O2	277.2183	277.2173	[M-H]-	195.1376;177.1281;135.0815	3.82	0.0045	0.0133	THA

*As the above metabolites were compiled separately for each brain region, there is overlap among the differential metabolites across different brain regions.

2. LC-MS Conditions for Nicotine and Its Metabolites

2.1. Chromatographic conditions:

The HPLC-MS/MS system consisted of an Acchrom.Tech S6000 ULTRR high-performance liquid chromatograph coupled with an AB SCIEX Triple Quad 6500+ triple quadrupole mass spectrometer equipped with an electrospray ionization (ESI) source. Separation was performed on a Waters ACQUITY UPLC BEH HILIC column (2.1 mm × 150 mm, 1.7 μm particle size). The column temperature was maintained at 40 °C. Mobile phase A consisted of 10 mM ammonium formate solution (pH 3.0), and mobile phase B was acetonitrile. The flow rate was 0.7 mL/min, the injection volume was 2 μL, and the total run time was 10 min. Data acquisition and processing were performed using SCIEX OS Analyst software. The gradient elution program is detailed in Table. S2.1.

Table S2.1. Gradient elution conditions for liquid chromatography.

Time（min）	Flow rate（mL/min）	mobile phase A（%）	mobile phase B（%）
0	0.7	8	92
2	0.7	14	86
2.1	0.7	30	70

5	0.7	30	70
5.1	0.7	40	60
7	0.7	40	60
7.1	0.7	8	92
10	0.7	8	92

2.2. Mass Spectrometry Conditions

Mass spectrometric detection was performed in multiple reaction monitoring (MRM) mode using an electrospray ionization (ESI) source operating in positive ion mode. The ESI source parameters were set as follows: ion spray voltage (IS): 5500 V; curtain gas (CUR): 50 psi; collision gas (CAD): 9; nebulizer gas (GS1): 40 psi; ion source temperature: 500 °C; heater gas (GS2): 40 psi. After parameter optimization, the MRM parameters for nicotine, its metabolites, and the corresponding internal standards are listed in Table. 2.2.

Table S2.2. MRM parameters for the target analytes and internal standards.

Compounds	Precursor ion （*m*/*z*）	Prroduct ion （*m*/*z*）	CE/V
Nicotine (Nic)	163.1	**130.2**	28
	163.1	132.2	20
Nic -d_3	166.1	**130.2**	28

Cotinine (Cot)	177.1	**80.0**	30
	177.1	98.1	30
Cot -d_3	180.0	**80.1**	30
3OH-Cot	193.1	**80.0**	35
	193.1	134.1	27
3OH-Cot -d_3	196.0	**80.1**	34
Nornicotine (NorNic)	149.0	**130.1**	23
	149.0	117.1	32
NorNic-d_4	153.1	**84.0**	38

Note: Product ions in bold font are used as quantifier ions.

2.3. Method Validation

Method validation experiments were conducted according to the instrument used and the different matrices. Taking brain tissue as an example, the linear ranges, limits of detection (LODs), and limits of quantification (LOQs) of the compounds are presented in Table. 2.3.

Table S2.3. Linear ranges, limits of detection, and limits of quantification of the compounds.

Compounds	Linear range（ng/mL）	Regression equation	Correlation coefficient（R^2）	Limit of detection（ng/mL）	Limit of quantitation（ng/mL）

NIC	0.2-500	y=0.0153x+0.00739	0.9978	0.97	3.20
COT	0.1-200	y=0.00897x+0.000996	0.9966	0.18	0.58
3-OHCOT	0.1-200	y=0.00665x+0.000315	0.9990	0.32	1.06
NNIC	0.125-125	y=0.0877x+0.00212	0.9982	0.35	1.15

2.4. The intra-day and inter-day precision and accuracy of the proposed method are presented in Table. 2.4.

Table S2.4. Intra-day and inter-day precision and accuracy of the method.

Compounds	Spiked concentration (ng/mL)	Intra-day			Inter-day		
		Determined concentration (ng/mL)	Accuracy/%	RSD/%	Determined concentration (ng/mL)	Accuracy/%	RSD/%
Nic	/	/	/	/	/	/	/
	10.0	9.12	90.90	7.17	10.28	102.80	7.69
	50.0	43.86	87.66	3.21	52.42	104.84	4.96

Cot	2.0	1.95	96.22	6.50	1.85	92.50	5.20
	10.0	8.90	88.80	3.11	9.25	92.50	6.45
	50.0	46.31	92.56	3.04	46.62	93.24	4.51
3OH-Cot	2.0	1.92	94.74	3.20	1.96	98.00	6.67
	10.0	8.60	85.75	6.87	9.58	95.80	6.30
	50.0	45.87	91.68	3.04	48.12	96.24	5.88
NorNic	2.0	2.18	100.07	4.07	1.78	89.00	7.74
	10.0	13.08	112.35	9.14	8.48	84.80	7.34
	50.0	58.06	106.15	3.21	42.76	85.52	5.16

2.5. The matrix effect, extraction recovery and stability of the proposed method are presented in Table. 2.5

5.

Table S2.5. Matrix effect, extraction recovery and stability of the method.

Compounds	Spiked concentration (ng/mL)	**Extraction Recovery (%) ± RSD**	**Matrix Factor (%)±** RSD	**Benchtop Stability (25°C, 6 h) RE (%)±** RSD	**Autosampler Stability (4°C, 24 h) RE (%)±** RSD	**Freeze-Thaw Stability (3 cycles) RE (%)** ± RSD

Nic	/	/	/			
	10.0	91.2±4.2	98.1±2.9	-3.2%±4.1	-4.1%±6.3	-5.8%±5.2
	50.0	93.5±3.1	101.4±3.2	-5.5%±2.8	-3.6%±2.9	-4.1%±4.0
Cot	2.0	86.7±4.8	94.8±4.1	-7.8%±3.5	-4.7%±5.1	-3.6%±3.7
	10.0	90.1±3.5	97.2±3.1	-3.9%±2.8	-2.8%±4.2	-9.2%±7.4
	50.0	92.8±2.4	99.6±2.7	-4.1%±6.3	-2.1%±3.6	-7.1%±5.9
3OH-Cot	2.0	84.2±5.1	92.5±4.5	-4.5%±4.8	2.3%±4.5	-6.3%±5.0
	10.0	88.9±3.8	95.8±3.2	-3.9%±4.0	1.5%±3.1	-2.0%±4.8
	50.0	91.6±2.9	98.3±2.6	-8.5%±7.8	-1.1%±2.7	-1.2%±3.6
NorNic	2.0	82.5±5.6	108.6±5.2	-5.7%±5.9	-6.2%±6.5	-11.3%±8.9
	10.0	87.3±4.2	105.4±3.9	-4.9%±4.7	-4.1%±5.0	-8.4%±6.8
	50.0	90.4±3.1	102.1±2.8	-1.2%±3.9	-3.5%±4.1	-7.2%±5.5

3. LC-MS Conditions for 13 neurotransmitters

3.1. Chromatographic conditions:

The HPLC-MS/MS system consisted of an Acchrom.Tech S6000 ULTRR high-performance liquid chromatograph coupled with an AB SCIEX Triple Quad 6500+ triple quadrupole mass spectrometer equipped with an electrospray ionization (ESI) source. Chromatographic separation was carried out on a Phenomenex Luna 3 µm PFP(2) 100 Å column (4.6 mm × 150 mm). The column temperature was maintained at 35 °C. Mobile phase A consisted of 0.1% formic acid in water, and mobile phase B was acetonitrile. The flow rate was 0.5 mL/min, the injection volume was 3 µL, and the total run time was 10 min with data acquisition from 1 to 8 min. Data acquisition and processing were performed using SCIEX OS Analyst software.The gradient elution program is detailed in Table 1.1.

Table S3.1. Gradient elution conditions for liquid chromatography.

Time（min）	Flow rate（mL/min）	mobile phase A（%）	mobile phase B（%）
0	0.5	100	0
1	0.5	80	20
5	0.5	10	90
6	0.5	10	90

7	0.5	100	0
10	0.5	100	0

3.2. Mass Spectrometry Conditions

Mass spectrometric detection was performed in multiple reaction monitoring (MRM) mode using an electrospray ionization (ESI) source operating in positive ion mode. The ESI source parameters were set as follows: ion spray voltage (IS): 5500 V; curtain gas (CUR): 50 psi; collision gas (CAD): 9; nebulizer gas (GS1): 40 psi; ion source temperature: 450 °C; heater gas (GS2): 40 psi. After parameter optimization, the MRM parameters for the neurotransmitter standards and internal standards are listed in Table. 3.2.

Table S3.2. MRM parameters for the target analytes and internal standards.

Compounds	Precursor ion （*m/z*）	Prroduct ion （*m/z*）	CE/V
Dopamine (DA)	154.1	**137.1**	14
	154.1	91	19
DA-d_4	158.1	**87**	13
Acetylcholine (ACH)	146.1	**60.1**	15
	146.1	98.1	30
ACH -d_4	150.1	**91**	15
3,4-	169.1	**152.0**	12

dihydroxyphenylacetic acid (DOPAC)			
	169.1	92	30
GABA	104.1	**87.1**	13.2
	104.1	43.2	20
GABA- d_2	106.1	**89.1**	12
NE	170.1	**107**	27
	170.1	124	16

Note: Product ions in bold font are used as quantifier ions.

3.3. Method Validation

Method validation experiments were conducted according to the instrument used and the different matrices. Taking brain tissue as an example, the linear ranges, limits of detection (LODs), and limits of quantification (LOQs) of the compounds are presented in Table. 3.3.

Table S3.3. Linear ranges, limits of detection, and limits of quantification of the neurotransmitters

Compounds	Linear range（ng/mL）	Regression equation	Correlation coefficient（R^2）	Limit of detection（ng/mL）	Limit of quantitation（ng/mL）

DA	0.1-100	y=0.09895x+0.0265	0.9988	0.05	0.17
ACH	0.5-100	y=0.06875x+0.00978	0.9989	0.25	0.84
3-MT	1-100	y=0.07789x+0.48066	0.9983	0.29	0.95
DOPAC	1-100	y=0.00285x+0.00072	0.9997	1.26	4.16
L-DOPA	0.2-50	y=0.06769x+0.00327	0.9986	0.31	1.02
5-HT	0.5-100	y=0.07879x+0.01591	0.9987	0.26	0.87
5-HTP	0.2-100	y=0.11613x+0.00408	0.9997	0.13	0.42
GABA	2-1000	y=0.00992x-0.00235	0.9997	0.09	0.31
GA	2-1000	y=0.01708x+0.00127	0.9990	0.13	0.44
Trp	1-1000	y=0.02648x+0.01579	0.9996	0.04	0.14
NE	1-100	y=000362x+0.00135	0.9986	1.64	5.4
E	0.5-50	y=0.00949x-0.00259	0.9968	0.45	1.5

5-HIAA	0.5-100	y=0.04586x-0.01384	0.9994	/	/

3.4. The intra-day and inter-day precision and accuracy of the proposed method are presented in Table. 2.4.

Table S3.4. Intra-day and inter-day precision and accuracy of the method.

Compounds	Spiked concentration (ng/mL)	Intra-day			Inter-day		
		Determined concentration (ng/mL)	Accuracy/%	RSD/%	Determined concentration (ng/mL)	Accuracy/%	RSD/%
DA	2.0	2.06	103	6.54	1.98	99	7.38
	20.0	21.2	106	7.22	19.4	97	8.44
ACH	2.0	1.78	89	6.51	1.97	98.5	6.78
	20.0	23.5	117.5	5.44	20.2	101	7.55
3-MT	2.0	1.85	92.5	5.56	1.89	94.5	6.84
	20.0	22	110	5.61	19.57	97.85	6.58
DOPAC	2.0	2.32	116	6.61	2.11	105.5	6.22

	20.0	20.8	104	5.88	20.4	102	6.10
L-DOPA	2.0	1.85	92.5	5.42	2.09	104.5	5.89
	20.0	17.3	86.5	6.86	19.4	97	7.52
5-HT	2.0	1.86	93	5.84	2.08	104	6.46
	20.0	22.4	112	5.42	20.4	102	6.82
5-HTP	2.0	2.09	104.5	4.25	1.89	94.5	5.94
	20.0	22	110	5.41	20.4	102	6.47
GABA	20	21.6	103.4	5.78	20.9	104.5	6.31
	200.0	186	93	4.36	205.0	102.5	5.88
GA	20.0	21.2	106	4.31	20.9	104.5	5.61
	200.0	228	114	5.45	187	93.5	7.05
Trp	20.0	21.2	106	4.58	19.6	98	5.04
	200.0	195	97.5	3.11	194	97	5.11
NE	2.0	1.70	85	4.24	1.84	92	6.17
	20.0	18.9	94.5	3.73	20.2	101	5.44
E	2.0	1.83	91.5	4.88	1.90	95	5.63
	20.0	19.8	99	3.57	21.1	105.5	5.14

3.5. The matrix effect, extraction recovery and stability of the proposed method are presented in Table. 3.5.

Table S3.5. Matrix effect, extraction recovery and stability of the method.

Compounds	Spiked concentration (ng/mL)	Extraction Recovery (%) ±RSD	Matrix Factor (%)±RSD	Benchtop Stability (25°C, 6 h) RE (%)±RSD	Autosampler Stability (4°C, 24 h) RE (%)±RSD	Freeze-Thaw Stability (3 cycles) RE (%)±RSD
DA	2.0	88.7±4.1%	112.3±4.5%	-2.8±6.5%	-1.8±4.6%	-4.9±7.8%
	20.0	91.2±4.3%	101.1±5.4%	-2.1±5.3%	1.5±7.2%	-3.8±6.4%
ACH	2.0	92.0±3.8%	87.1±7.5%	-1.8±4.6%	1.0±5.9%	-3.3±5.5%
	20.0	87.3±6.2%	86.0±9.6%	1.5±7.2%	0.7±5.0%	-2.6±8.9%
3-MT	2.0	88.1±5.4%	103.8±3.1%	1.5±7.2%	-6.2±7.9%	-1.9±7.1%
	20.0	89.0±4.6%	105.2±2.8%	1.0±5.9%	-5.0±6.5%	-1.5±6.0%
DOPAC	2.0	96.1±4.1%	90.8±9.1%	-0.7±5.0%	-4.4±5.5%	-7.8±9.1%
	20.0	98.3±3.3%	92.9±7.3%	-6.2±7.9%	-0.4±9.0%	-6.5±7.6%

L-DOPA	2.0	88.5±5.0%	91.1±6.1%	-5.0±6.5%	-7.5±8.6%	-5.8±6.4%
	20.0	91.2±4.6%	96.2±4.2%	-4.4±5.5%	-6.1±7.0%	-2.0±10.5%
5-HT	2.0	87.5±4.4%	97.0±3.8%	-0.4±9.0%	3.5±6.8%	-1.5±8.5%
	20.0	89.3±77.5%	91.2±6.9%	-1.1±7.4%	-2.8±5.6%	-1.2±7.0%
5-HTP	2.0	94.8±33.5%	92.8±5.2%	-7.5±8.6%	2.8±5.6%	-7.8±9.1%
	20.0	96.0±2.8%	87.5±7.2%	-6.1±7.0%	2.3±4.8%	-6.5±7.6%
GABA	20	96.8±2.3%	89.2±5.8%	-5.4±6.0%	-3.9±6.0%	-5.8±6.4%
	200.0	88.9±4.6%	88.4±4.9%	-3.5±6.8%	-3.2±4.9%	-2.0±8.5%
GA	20.0	92.5±4.0%	95.8±3.7%	2.8±5.6%	-2.7±4.1%	-1.5±8.5%
	200.0	95.8±2.6%	94.7±3.1%	-2.3±4.8%	-5.4±6.3%	-1.2±7.0%
Trp	20.0	94.5±5.4%	87.4±9.8%	-3.9±6.0%	-6.8±8.2%	-9.8±8.5%
	200.0	96.3±2.4%	86.8±7.9%	-3.2±4.9%	-5.5±6.7%	-8.3±8.1%
NE	2.0	88.1±44.8%	91.8±4.5%	2.2±6.0%	-4.8±5.7%	-7.4±6.9%
	20.0	90.0±3.9%	93.0±6.4%	1.8±5.1%	-1.0±8.5%	-1.0±8.1%
E	2.0	91.8±4.2%	91.1±6.6%	-5.5±6.7%	-8.5±6.9%	-5.5±7.3%
	20.0	94.2±2.8%	84.1±6.0%	-4.8±5.7%	-7.1±7.4%	-4.4±5.9%
